# Oxazolochlorins 21. Most Efficient Access to *meso*-Tetraphenyl- and *meso*-Tetrakis(pentafluorophenyl)porpholactones, and Their Zinc(II) and Platinum(II) Complexes

**DOI:** 10.3390/molecules25184351

**Published:** 2020-09-22

**Authors:** Damaris Thuita, Dinusha Damunupola, Christian Brückner

**Affiliations:** Department of Chemistry, University of Connecticut, Unit 3060, Storrs, CT 06269–3060, USA; damaris.thuita@uconn.edu (D.T.); dinudamunupola@uconn.edu (D.D.)

**Keywords:** porphyrin, porphyrinoids, porpholactone, oxidation

## Abstract

*meso*-Phenyl- and *meso*-pentafluorophenyl-porpholactones, their metal complexes, as well as porphyrinoids directly derived from them are useful in a number of technical and biomedical applications, and more uses are expected to be discovered. About a dozen competing and complementary pathways toward their synthesis were reported. The suitability of the methods changes with the *meso*-aryl group and whether the free base or metal derivatives are sought. These circumstances make it hard for anyone outside of the field of synthetic porphyrin chemistry to ascertain which pathway is the best to produce which specific derivative. We report here on what we experimentally evaluated to be the most efficient pathways to generate the six key compounds from the commercially available porphyrins, *meso*-tetraphenylporphyrin (TPP) and *meso*-tetrakis(pentafluorophenyl)porphyrin (T^F^PP): free base *meso*-tetraphenylporpholactone (TPL) and *meso*-tetrakis(pentafluorophenyl)porpholactone (T^F^PL), and their platinum(II) and zinc(II) complexes TPLPt, T^F^PLPt, TPLZn, and T^F^PLZn, respectively. Detailed procedures are provided to make these intriguing molecules more readily available for their further study.

## 1. Introduction

**Scheme 1:**
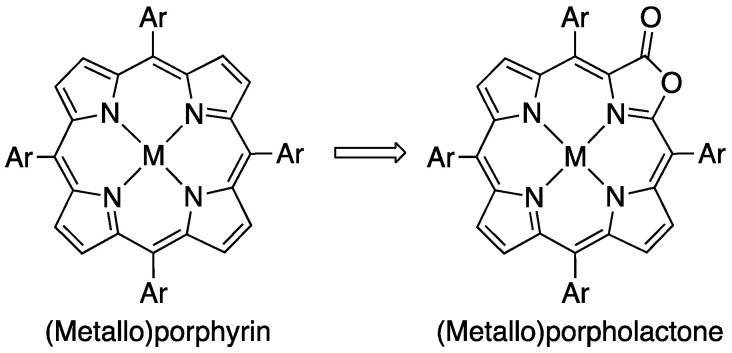
Generalized formation of *meso*-arylporpholactones from *meso*-arylporphyrins.

Porpholactones, members of the family of so-called pyrrole-modified porphyrins [1], are porphyrinoids in which a ß,ß’-bond of the parent porphyrin was replaced by a lactone moiety. From the perspective of the tetrapyrrolic structure of porphyrins, a pyrrolic building block was replaced by an oxazolidone (Scheme 1).

**Scheme 2:**
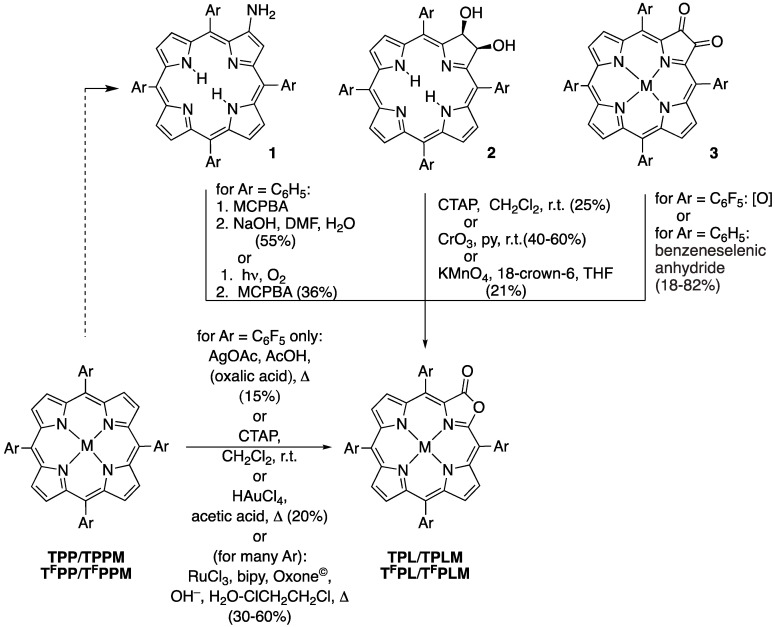
Select literature-known reaction conditions toward the formation of *meso*-aryl(metallo)porpholactones. TPP = *meso*-tetraphenylporphyrin; TPPM = [*meso*-tetraphenylporphyrinato] metal complex; T^F^PP = *meso*-tetrakis(pentafluorophenyl)porphyrin; T^F^PPM = [*meso*-tetrakis(pentafluorophenyl)porphyrinato] metal complex; TPL = *meso*-tetraphenylporpholactone; TPPM = [*meso*-tetraphenylkporpholactonato] metal complex; T^F^PL = *meso*-tetrakis(pentafluorophenyl)porpholactone; T^F^PLM = [*meso*-tetrakis(pentafluorophenyl)porpholactonato] metal complex.

The historically first porpholactone, *meso*-tetraphenylporpholactone (TPL) was derived from *meso*-tetraphenylporphyrin (TPP) [2]. While several phenyl- and substituted phenyl-derivatives were reported [3,4], the by far most popular derivative is the *meso*-tetrakis(pentafluorophenyl)porpholactone (T^F^PL) and its metal complexes (T^F^PLM) [1,5,6]. This is because of their relative ease of synthesis in one step from T^F^PP using a number of different methodologies (see below Scheme 2) and the multiple possibilities to derivatize the C_6_F_5_-groups by means of nucleophilic aromatic substitution reactions to, for example, render them water-soluble [6,7,8]. The presence of a ß,ß’-lactone moiety in carbaporphyrins [9], thiaporphyrins [10,11], octaalkylporphyrins [12], or subporphyrins [13] is known but much more rare and the utility of these compounds has not yet been shown.

Upon the replacement of one or two ß,ß’-bond(s) of the parent porphyrin by lactone moieties, the electronic structure of the chromophore is altered, sometimes in profound ways, even though the UV-vis spectra of free base TPP and TPL are surprisingly similar to each other [4]. The electronic influence of the lactone moieties on the chromophore was studied in some detail [4,5,6,14,15,16]. On account of only a minor steric interaction between the carbonyl oxygen and the *o*-hydrogen/fluorine atoms on a neighboring *meso*-aryl group, the introduction of one (or even two) lactone moieties alters the idealized planar conformation of the parent porphyrins only in subtle ways [4,14,16,17,18].

Like their porphyrin congeners [19], both TPL and T^F^PL are competent ligands for a wide range of metals, forming the metal complexes TPLM and T^F^PLM, respectively (with M = Mg^II^, Ni^II^, Mn^III^, Fe^III^, Zn^II^, Y^III^, Ag^II^, Pd^II^, Pt^II^, Yb^III^) [4,5,6,20,21,22,23]. The metal ions may already be present in the metalloporphyrin/chlorin starting materials or are inserted later into the free base macrocycle.

*meso*-Arylporpholactones and their metal complexes have found utility as, for instance, olefin epoxidation [24], hydroformylation [25], nitrogen transfer [23], sulfoxidation [26], or electrochemical hydrogen evolution reaction catalysts [27,28], or as photocatalysts for oxidative C-H functionalizations [29]. Other porpholactones found uses as chlorophyll models [14] or as bioinorganic models for intermediates in the catalytic cycles of heme-based peroxidases, catalases, and oxidases [3]. Porpholactones are better lanthanide sensitizers than porphyrins [21] and served as bioimaging [21] or phototheranostic agents [30], oxygen-sensing dyes in pressure-sensitive paints [31,32,33], optical cyanide sensors [7,34], or high-pH sensors [35,36], including as ratiometric dye suitable for the full-field optical mapping of the pH of concrete surfaces [36]. T^F^PL-based photosensitizers also exhibit higher lipoprotein binding affinity, cellular uptake, and intracellular localization selectivity when compared to the parent porphyrins [37].

Multiple distinct pathways of converting (metallo)porphyrins to (metallo)porpholactones were described in nearly two dozen individual papers. Some were specifically developed to provide porpholactones, others became the source of porpholactones by serendipity (Scheme 2) [1,6]. For all methods, the exact mechanisms of the formation of porpholactones are not known. It appears, however, that (metallo)porpholactones are generally a thermodynamic sink in the oxidative degradation pathways of many ß-substituted (metallo)-porphyrins/chlorins. Simple one-step reactions that generate a porpholactone directly from a porphyrin are generally burdened by the need to isolate the porpholactone from unreacted porphyrin (of similar R_f_ value as the porpholactone) and among several other (over)oxidation products [5,38]. In addition, not all conditions are applicable to all *meso*-arylporphyrins, or were tested for their applicability to varying aryl groups. On the other hand, the oxidation of several ß-substituted (metallo)-porphyrins (**1**, **3**) and -chlorins (**2**) converted them to porpholactones in more rational and sometimes even selective fashion [3,4], facilitating the isolation of the product of relative low polarity from the starting materials of (much) higher polarity. Alas, some of the reactions are inefficient or the starting materials require a multi-step synthesis from the corresponding porphyrins [2,3,4,39]. Except for the MnO_4_^–^-oxidations of diol chlorin **2**, a reaction specifically developed for the synthesis of porpholactones [4], none of the reactions were shown to possess some generality with respect to the *meso*-aryl group or a central metal ion present.

As the number of inquiries from non-porphyrin chemist practitioners we received over the years clearly highlighted to us, the many known methods and pathways toward porpholactones present a hard to navigate maze for the non-specialist. A decision as to which option is the most expedient, safe, simple, or economical is not readily possible. There appears to be no commercial source for any of the porpholactones.

Over the course of the past decade [40], we developed and optimized our own procedures or evaluated numerous syntheses published by others toward the synthesis of free base TPL/T^F^PL, and their zinc(II) and platinum(II) complexes. In this contribution, we like to delineate in detail the most efficient paths toward TPL/TPLZn/TPLPt and T^F^PL/T^F^PLZn/T^F^PLPt we are aware of, with the aim of providing the non-specialist access to these intriguing and versatile materials. The procedures spelled out (see Supporting Information) were derived from published methods developed by us [4], or the research group of Zhang [41], and tested multiple times in our laboratories. The detailed procedures incorporate hitherto unpublished practical details.

## 2. Results and Discussion

Since the most efficient pathways toward the *meso*-phenyl- and *meso*-pentafluorophenyl-substituted porpholactones vary, we will discuss the syntheses of these two families separately.

### 2.1. The Most Efficient Pathway toward T^F^PL/T^F^PLZn/T^F^PLPt

T^F^PP is readily synthesized [42] or is commercially available from a variety of supply houses, as are its metallated derivatives T^F^PPM (for M = Zn^II^ or Pt^II^) [43,44]. Their better solubility renders the fluorinated derivatives to be a great platform for many modification reactions, including their one-step conversion to the corresponding (metallo)porpholactones T^F^PL/T^F^PLM. We found the RuCl_3_/Oxone^®^-mediated oxidation pathway, as described by Zhang and co-workers [41], to be the most efficient pathway to generate free base T^F^PL as well as T^F^PLMs (for M = Zn^II^ or Pt^II^) by oxidation of the corresponding porphyrin metal complexes T^F^PPM (for M = Zn^II^ or Pt^II^). The reaction was described to be more suitable for substrates bearing electron-withdrawing *meso*-substituents [41].

The ruthenium-catalyzed oxidation is a one-step, one pot process in a biphasic mixture of ClCH_2_CH_2_Cl (1,2-dichloroethane) and H_2_O/NaOH in which the oxidant mixture RuCl_3_/bipy/Oxone^®^ is heated with the starting (metallo)porphyrin (dissolved in the organic phase) under reflux conditions [41]. Depending on the reactivity of the substrate, the reaction allows a convenient variation of the oxidant:substrate stoichiometry, as well as reaction times. The reaction is robust to minor deviations in the reaction conditions. Even though several products may be formed that require their chromatographic separation, under the optimized conditions, the reaction is simple, reasonably fast, clean, and comparatively high-yielding.

Under the standard ruthenium-catalyzed oxidation conditions reported by Zhang and co-workers (0.5 eq RuCl_3_, 0.5 eq bipyridine, 5 eq Oxone^®^, 5 eq NaOH, 5 h reaction time), about 60–75% T^F^PP converted, yielding multiple products. The major product is chromatographically isolated free base T^F^PL (~30% isolated yield at a 500 mg of T^F^PP scale, R_f_ = 0.52 (silica/CH_2_Cl_2_)), along with a mixture of dilactones (in 10–15% yield, R_f_ = 0.31 (silica/CH_2_Cl_2_)) but that are readily separated, as is the unreacted T^F^PP (R_f_ = 0.76 (silica/CH_2_Cl_2_)). Increasing the oxidant stoichiometry in combination with extended refluxing times led to full conversion of the porphyrin and an increase of the yield of T^F^PL to up to 45% (at a 200 mg of T^F^PP scale), with the remainder made up by the dilactone mixture. See Supporting Information for a detailed procedure and reproduction of the key spectra of all products.

**Scheme 3:**
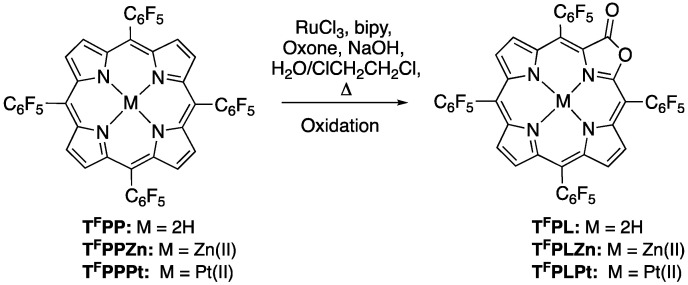
The synthesis of *meso*-tetrakis(pentafluorophenyl)porpholactone (T^F^PL) and its zinc(II) (T^F^PLZn) and platinum(II) (T^F^PLPt) complexes.

Metallation of T^F^PP generally increases its susceptibility towards the Ru-mediated oxidation [41]; accordingly, the formation of the corresponding T^F^PLM is faster and higher yielding in comparison to the formation of their free base congener [41]. Thus, the oxidation of the metalloporphyrins T^F^PPZn and T^F^PPPt to obtain the corresponding metalloporpholactones T^F^PLZn/Pt (Scheme 3) is much preferred over the insertion of zinc(II)/platinum(II) into free base T^F^PL, although the latter is also an option [4,45].

Under the standard oxidation conditions (1 eq T^F^PPM, 0.5 eq RuCl_3_, 5 eq Oxone^®^, 0.5 eq bipyridine, 5 eq NaOH, 5 h reaction time), T^F^PPZn provided T^F^PLZn in 35% yield (at a scale of 500 mg of T^F^PPZn). The yield can be increased to 50–60% when the initial concentrations of T^F^PPZn and RuCl_3_ are doubled along with a corresponding increase of oxidant (10 eq Oxone^®^, 10 eq NaOH) and using extended reaction times (up to 72 h). In both cases, a mixture of dilactone isomers is observed to be the major byproduct (in yields of 15–20% under the standard oxidation conditions). T^F^PLPt was produced in ~50% yield from T^F^PPPt under the standard conditions (at a scale of 100 mg of T^F^PPPt) and, again, along with the dilactone mixture as the primary byproduct (~10–15%). Increase of the ratio of oxidant to 15 eq and reaction times up to 48 h increases the yield of T^F^PLPt substantially (up to 75%), along with an increased yield of the dilactone mixture (20%). For both metalloporpholactones, preparative plates (500 µm silica gel, 20 × 20 cm plates) can be conveniently used for the isolation of the product in small scale reactions (40–50 mg). Larger scale reactions were purified using column chromatography. The products are identified based on their diagnostic UV-vis, ^1^H NMR, and IR spectroscopy or mass spectrometry data (see ESI).

None of the other direct oxidation methods (using MnO_4_^–^ [17]; Ag(I) [5], or Au(III) [46]) could compete in terms of yields, scalability, and ease of separation of the desired products with the RuCl_3_/Oxone^®^/base methodology [41]. While a two-step method analogous to that described below for the syntheses of the nonfluorinated derivatives TPL/TPLM works well for the synthesis of T^F^PL/T^F^PLM [4,17], it is unnecessarily arduous and only worth pursuing when selectively dilactone derivatives are desired [17].

### 2.2. The Most Efficient Pathway toward TPL/TPLZn/TPLPt

Porphyrin TPP is readily synthesized in multi-gram scales [47,48,49] and, modestly priced [43]. Its zinc(II) complex TPPZn is readily formed [19]. While the corresponding platinum(II) complexes TPPPt can also be formed [45,50], its low solubility in most solvents make it nearly intractable and unsuitable as a starting materials for the preparation of TPLPt using any of the available methods. Therefore, TPLPt needs to be prepared by the platinum(II) insertion into the free base porpholactone TPL using either conventional thermal [50] or microwave-assisted [45] methodologies.

The one-step RuCl_3_-mediated oxidation of the comparably electron-rich TPP is less efficient (isolated yields of 10–30% of TPL at a 200 mg scale) compared to the oxidation of the fluorinated analogue T^F^PP [41], thus requiring larger stoichiometric ratios of the oxidant as well as longer reaction times (up to one week, or more). In our hands, the isolation of the porpholactone at any larger scale from its side products proved also to be non-trivial. Neither TPP nor TPPZn are susceptible to the MnO_4_^–^- [17], or Ag(I)-mediated [5] oxidation protocols. The Au(III)-mediated oxidation of the silver(II) complex of TPP forms the free base TPL in 23% yield [46], but this route is also not truly competitive (and the oxidant costly). Thus, access to the porpholactone TPL is best achieved along a multi-step processes, with access to TPLZn/Pt by means of zinc(II) and platinum(II) insertions into the free base TPL, respectively.

Among the possible multi-step processes toward TPL (Scheme 1), one process stands out as particularly suitable for its simplicity and high yields: We have reported in detail [51,52], both the OsO_4_-mediated *cis-vic*-dihydroxylation, followed by a diol oxidation of the intermediate dihydroxychlorin [4,17,53,54]. However, there a number of variabilities in these processes: The intermediate osmate ester can be isolated and oxidized, or the osmate ester is reduced to a diol and is then oxidized, with a number of possible oxidants for either step (MnO_4_^–^ with the counter cations cetyltrimethyl ammonium (CTAP) or [18-C-6]·K^+^ [4,53]; CrO_3_ [17,54]).

**Scheme 4:**
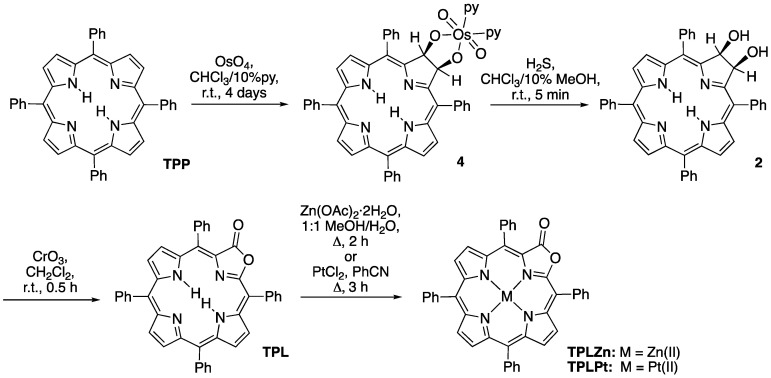
The most efficient synthesis of *meso*-tetraphenylporpholactone (TPL) and its zinc(II) (TPLZn) and platinum(II) (TPLPt) complexes.

The most simple, scalable and highest yielding process we find is the osmylation of TPP (performed in up to a 5 g scales), the isolation of osmate ester **4**, followed by its reductive cleavage using gaseous H_2_S, to form chlorin diol **2** [51], and finally a CrO_3_-mediated oxidation of the diol (performed in up to 300 mg scales) (Scheme 4) [17,54]. The overall yield of 45% of TPL over all three steps from TPP is good (even without considering that ~20% of TPP is recovered after the first step), the reaction is scalable, straight-forward, and well-controlled.

In detail, the synthesis of TPL involves first, an OsO_4_-mediated dihydroxylation step to yield the intermediate osmate ester **4** that is isolated and subsequently reduced to diol **2** by a purge of H_2_S gas. Isolated polar product **2** is then oxidized to TPL using CrO_3_ in the presence of pyridine over 30 min under ambient conditions; this produced the readily isolable non-polar bright pink compound TPL (in 35–40% overall isolated yield). The use of a 20 × 20 cm, 500 µm silica gel preparative chromatography plate for scales up to 50 mg diol is convenient; above this column chromatography is advisable. We observed that the oxidation shows best results when run in small batches of 250–300 mg of **2**; the reaction appears to be retarded at scales of 500 mg, or more. Diagnostic UV-vis, ^1^H NMR, and IR spectroscopy or mass spectrometry data can be used to unequivocally identify the product TPL and its metal complexes. See Supporting Information for a detailed procedure and a reproduction of the key spectra of all products and intermediates.

Metal insertions into free base TPL prepare the desired metal complexes [4]. See Supporting Information for a detailed procedure and a reproduction of the key spectra. While the zinc(II) complex TPLZn can, in principle, also be prepared from the soluble porphyrin zinc complex TPPZn, the isolation of the intermediate chlorin is easier for the free base (the metallochlorin is significantly more polar and ‘streaks’ during chromatographic separation). Also, the diol oxidation of the chlorin diol zinc complex is characterized by lower yields that the corresponding free base chlorin diol oxidation.

The biggest drawback of this multi-step method toward TPL/TPLM is the use of the toxic reagents OsO_4_, H_2_S, and CrO_3_. The risk of working with OsO_4_ can be minimized by scaling the osmylation reactions such that entire 250 mg, 500 mg, or 1 g ampules of OsO_4_ can be used; they can be opened (fume hoods, hand and eye protection needed) and immediately be drowned (cap and body) in the reaction mixture that is then stoppered and left to react. Before the reactions are worked up, the glass vials are retrieved. The use of H_2_S and CrO_3_ can be circumvented by oxidation of the osmate ester **4** with CTAP (or KMnO_4_/18-C-6) [53]. However, the yields for these reactions are slightly lower (25–30%), particularly at larger (> 250 mg diol osmate ester) scales. The reaction is hard to control and often leads to ‘overoxidation’. Practical difficulties in the isolation of the product resulting from the formation of the hard-to-remove sludges the CTAP side products form also contribute to the lower isolated yields at larger scales. Lastly, using the osmate ester **4** as substrate, the fate of the osmium is unclear upon MnO_4_^–^-oxidation [35,52]. Thus, given the risks involved with re-generating OsO_4_ unwittingly, these oxidations require also great care, mitigating some of the advantages of avoiding CrO_3_.

## 3. Materials and Methods

### 3.1. Materials

All solvents and reagents (Sigma-Aldrich, St. Louis, MO, USA and Acros, Fair Lawn, NJ, USA) were used as received. T^F^PP [42] was either synthesized according to the literature procedure or obtained commercially [43,44]. T^F^PPZn [42] and T^F^PPPt [20] were synthesized by metal insertion into the free base according to literature procedures. TPP was synthesized according to a literature procedure [47], and it is also commercially readily available [43,44].

All solvents were reagent-grade, or better, and were used as received. The CHCl_3_ used was EtOH-stabilized (amylene-stabilized CHCl_3_, for example, is unsuitable for the osmylations).

Aluminum-backed, silica gel 60, 250-μm thickness analytical plates were used for analytical TLC; Either 20 × 20 cm, glass-backed, silica gel 60, 500 µm thickness preparative TLC plates or standard grade, 60 Å, 32–63 µm flash column silica gel were used for the preparative chromatography.

Detailed procedures with spectroscopic data and reproduction of the key spectra of the products and intermediates (were present) are provided in the ESI.

### 3.2. Two-Step Procedure for Synthesis of meso-Tetraphenyl-2-Oxa-3-Oxoporphyrin (TPL) by OsO_4_-Mediated Dihydroxylation, Followed by CrO_3_ Oxidation

#### 3.2.1. Dihydroxylation of TPP; Synthesis of *meso*-Tetraphenyl-2,3-*cis*-dihydroxychlorin **2**

To a 500 mL round-bottom flask equipped with a stir bar, *meso*-tetraphenylporphyrin (TPP) (2.20 g, 3.58 mmol) was dissolved in a mixture of CHCl_3_ (270 mL, EtOH-stabilized) and freshly distilled pyridine (30 mL). A glass ampoule of 1.0 g of OsO_4_ (1.0 g, 3.93 mmol, ~1.1 equiv.) was opened and immediately submerged (cap and body) into the mixture. The flask was then stoppered, shielded from light with aluminum foil, and stirred at ambient temperature (20–24 °C) The progress of the reaction was monitored by occasional TLC. Once no further progress was noted (after about 4 days), the solvent was removed on a rotary evaporator as best as possible. The crude osmate ester product was then dissolved in a solution of 10% MeOH/CHCl_3_ (~250 mL). The osmate ester was then reductively cleaved by purging with gaseous H_2_S for 5 min (Note 2); the mixture was stirred for an additional 30 min before the majority of the excess H_2_S was purged from the solution by a stream of nitrogen gas through the solution. Following the addition of further MeOH (20 mL), the precipitated black OsS was filtered off through a short plug of Celite^®^. The filtrate was evaporated to dryness by a stream of N_2_ or rotary evaporation. The resulting residue was dissolved in a minimal amount of CHCl_3_ and loaded onto a silica gel column (25 × 7 cm) and eluted with CH_2_Cl_2_. The first fraction was starting material TPP (250 mg, 11% recovery). CH_2_Cl_2_/1.5% MeOH then eluted diol chlorin **2**. Slow evaporation from a MeOH/CH_2_Cl_2_ mixture provided product **2** as a bright purple crystalline material (1.00 g, 1.40 mmol, 49% yield). R_f_ (silica−CH_2_Cl_2_/1.5% MeOH) = 0.68.

All reaction steps, including the solvent removal involving the H_2_S-exposed solutions, were performed in a fume hood following usual laboratory safety practices (gloves, eye protection and lab coats). Caution: Note the hazard and risk of using OsO_4_ (acute oral and inhalation toxin, skin irritant, serious danger to eye damage). Further, note the hazard and risk of using H_2_S (fatal if inhaled, extended exposure reduces the ability to smell the gas; flammable). A fume hood is essential. Trap all excess H_2_S (bleach traps, for example).

#### 3.2.2. Synthesis of *meso*-Tetraphenyl-2-oxa-3-oxoporphyrin (TPL) via CrO_3_ Oxidation of Diol **2**

In a 25 mL round-bottom flask equipped with a stir bar and two carbon pellets, *meso*-tetraphenyl-2,3-*cis*-dihydroxychlorin **2** (100 mg, 0.154 mmol) was dissolved in pyridine (5 mL). Chromium trioxide CrO_3_ (∼10 equiv., 154 mg, 1.54 mmol) was added to the solution. The round-bottom flask was shielded from light with aluminum foil and stirred at ambient temperature. The disappearance of the starting material and the appearance of the product were monitored by TLC. Once no further progress of the reaction was detectable (after ∼30 min), CH_2_Cl_2_ (25 mL) was added and the mixture was transferred into a 125 mL separatory funnel and washed with water (3 × 25 mL). The organic phase was separated and filtered through a short plug of diatomaceous earth (Celite^®^) and dried over anhyd. Na_2_SO_4_. The drying agent was removed by gravity filtration and the filtrate was evaporated to dryness by rotary evaporation. A gentle stream of N_2_ passed through the crude material for several h ensured that the crude material was thoroughly dried. The crude material was dissolved in a few mL CH_2_Cl_2_ coated onto silica. The material was then dry-loaded onto a flash column (~6 × 25 cm) silica–CH_2_Cl_2_) and chromatographed to provide TPL in 40–60% yield (60 mg) as a pinkish red solid. R_f_ (silica–CH_2_Cl_2_) = 0.67.

All reaction steps described below, except for the solvent removal, were performed in a fume hood following usual laboratory safety practices (gloves, eye protection and lab coats). Caution: CrO_3_ is a strongly oxidizing solid, toxic if swallowed, causes severe skin burns, is mutagenic, and a reproductive toxin. Eye protection and gloves needed when handling this chemical.

### 3.3. Preparation of Metalloporpholactone TPLZn via Zinc Insertion into Free Base TPL

A 250 mL round-bottom flask was equipped with a stir bar, heating mantle, and a reflux condenser. The flask was charged with porpholactone TPL (123 mg, 1.94 mmol) and Zn(OAc)_2_·2H_2_O (123 mg, 0.193 mmol, 3 equiv.) dissolved in CHCl_3_/MeOH (8:1, 80 mL). The reaction mixture was heated to reflux for ~2 h. Upon completion of reaction as observed by TLC, the reaction mixture was cooled to room temperature and filtered through Celite^®^. The filtrate was concentrated, the concentrate was loaded onto the silica gel column, and purified by short column chromatography (silica, CH_2_Cl_2_) to produce TPLZn as a purple-colored film. Recrystallization by dissolution in a few mL of CH_2_Cl_2_, addition of ~50% MeOH, and slow removal of the CH_2_Cl_2_ on a rotary evaporator (at ambient T) precipitated TPLZn; it was retrieved by filtration and air-dried to provide TPLZn as a bright purple crystalline solid in 97% yield (130 mg, 0.143 mmol). R_f_ (silica–CH_2_Cl_2_) = 0.44.

All steps, except for the solvent removal, were performed in a fume hood following usual laboratory safety practices (gloves, eye protection and lab coats).

### 3.4. Preparation of Metalloporpholactone TPLPt via Platinum Insertion into Free Base TPL

A 50 mL round-bottom flask was equipped with a stir bar, heating mantle, a reflux condenser and a N_2_ inlet. TPL (65.0 mg, 0.086 mmol) was dissolved in benzonitrile PhCN (5 mL) and added to a refluxing solution of benzonitrile PhCN (20 mL) and PtCl_2_ (61 mg, 0.344 mmol, 4 equiv.). The mixture was heated to reflux for 3 h. When the starting material was consumed (reaction monitored by TLC), the reaction mixture was allowed to cool and was evaporated to dryness by rotary evaporation. The resulting mixture was dissolved in CH_2_Cl_2_ (2–5 mL), loaded as solution onto a flash column (silica-CH_2_Cl_2_) and chromatographed. TPLPt was isolated in 71% (50 mg) yield as a magenta powder. R_f_ (silica-CH_2_Cl_2_) = 0.17.

All steps, except for the solvent removal, were performed in a fume hood following usual laboratory safety practices (gloves, eye protection and lab coats).

### 3.5. One-Step Synthesis of meso-Tetrakis(pentafluorophenyl)-2-oxa-3-oxoporphyrin T^F^PL by RuCl_3_/Oxone^®-^Mediated Oxidation. General Procedure

In a 750 mL round-bottom (or a two-neck, round-bottom) flask equipped with a stir bar and reflux condenser, T^F^PP (0.8 g, 0.8 mmol, 1 equiv.) and RuCl_3_ (85 mg, 0.4 mmol, 0.5 equiv.) were dissolved in 1,2-dichloroethane (ClCH_2_CH_2_Cl, 250 mL). A solution containing 2,2′-bipyridine (65 mg, 0.4 mmol, 0.5 equiv.) in ClCH_2_CH_2_Cl (7 mL) and water (250 mL) were added. A condenser was attached to one of the necks and the solution was heated to reflux. A mixture of Oxone^®^ (7.4 g, 12 mmol, 15 equiv.) and NaOH (480 mg, 12 mmol, 15 equiv.) in water (15 mL) was added periodically in five equal portions over the course of 5 h to the refluxing solution. After completion of the addition, the reaction mixture was refluxed overnight. The mixture was allowed to cool to room temperature, quenched with a saturated aqueous solution of Na_2_S_2_O_3_ (2.0 g in 5 mL of water). The reaction mixture was stirred, allowed to stand for 30 min, and transferred to a 750 mL separatory funnel. The (bottom) organic layer was separated, and the aqueous layer was extracted twice using CH_2_Cl_2_ (2 × 150 mL). The combined organic fractions were washed with a saturated aqueous NaCl solution (2 × 150 mL). The organic layer was dried with anhydrous Na_2_SO_4_ (30 min) and the drying agent was removed by gravity filtering. The filtrate was reduced to dryness using rotary evaporation (in a 500 mL round-bottom flask). The residue was dissolved in CH_2_Cl_2_ (2–5 mL), loaded as a solution onto a packed column (silica, 5 × 60 cm, with a fritted disk, eluent: CH_2_Cl_2_/hexanes = 2:1) and separated to obtain the product T^F^PL as a purple solid upon solvent removal in 30% yield (240 mg, 0.24 mmol). R_f_ (silica–CH_2_Cl_2_/20% hexane) = 0.68.

All steps, except for the solvent removal, were performed in a fume hood following usual laboratory safety practices (gloves, eye protection and lab coats). CAUTION: 1,2-Dichloroethane is harmful if swallowed, causes skin and eye irritation, toxic if inhaled, and is considered as a potential carcinogen. Flammable with a flash point of 13 °C.

### 3.6. Synthesis of [meso-Tetrakis(pentafluorophenyl)-2-oxa-3-oxoporphyrinato]zinc(II) (T^F^PLZn)

Prepared in 35% yield (189 mg) as a bright purple powder according to the general procedure for the synthesis of T^F^PL synthesis using T^F^PPZn (0.52 g, 0.5 mmol, 1 equiv.) and RuCl_3_ (53 mg, 0.25 mmol, 0.5 equiv.) in ClCH_2_CH_2_Cl (150 mL) and water (150 mL), respectively in a 500 mL round-bottom flask equipped with a stir bar was mixed with a solution of 2,2′-bipyridine (40 mg, 0.25 mmol, 0.5 equiv.) in ClCH_2_CH_2_Cl (5 mL). A mixture of Oxone^®^ (3.1 g, 5 mmol, 10 equiv.) and NaOH (290 mg, 5 mmol, 10 equiv.) in water (15 mL) was added periodically over 5 h in equal portions. TLC control. Workup as per general procedure. R_f_ (silica–CH_2_Cl_2_/20% hexanes) = 0.48.

### 3.7. Synthesis of [meso-Tetrakis(pentafluorophenyl)-2-oxa-3-oxoporphyrinato]platinum(II) (T^F^PLPt)

Prepared in 50% yield (191 mg) as a reddish powder according to the general procedure described for the synthesis of T^F^PL synthesis using T^F^PPPt (100 mg, 0.16 mmol, 1 equiv.) and RuCl_3_ (16.6 mg, 0.08 mmol, 0.5 equiv.) in ClCH_2_CH_2_Cl (50 mL) and water (50 mL) in a 250 mL round bottom flask, mixed with a solution of 2,2′-bipyridine (12.5 mg, 0.08 mmol, 0.5 equiv.) in ClCH_2_CH_2_Cl (1.5 mL). A mixture of Oxone^®^ (243.5 mg, 1.6 mmol, 10 equiv.) and NaOH (64.0 mg, 1.6 mmol, 10 equiv.) in water (5 mL) was added in five portions over 5 h while the solution was refluxed for 8 h. Work up and isolation of product were carried out according to the general procedure. R_f_ (silica–CH_2_Cl_2_/20% hexanes) = 0.52.

## 4. Conclusions

The most efficient pathways toward the most popular free base porpholactones, TPL and T^F^PL, and their zinc(II) and platinum(II) complexes TPLZn/TPLPt and T^F^PLZn/T^F^PLPt, respectively, are described. The pathways differ and are dependent on the substrate reactivity and solubility: The RuCl_3_/Oxone^®^ mediated oxidation of T^F^PP/T^F^PPM is the preferred pathway toward the *meso*-pentafluorophenylporpholactones T^F^PL/T^F^PLM. The metalloporphyrins are suitable starting materials for the direct formation of metalloporpholactones. However, a multi-step process is best for the non-fluorinated derivatives TPL and subsequent metal insertion into the free base porpholactone provides access to the metal complexes TPLZn/TPLPt. A number of variations for the multi-step process are available, depending on the comfort level of the researchers working with H_2_S and/or CrO_3_. The preparation of multi-100 mg quantities of all (metallo)porpholactones in acceptable yields can be achieved in predictable ways from commercially available materials, but chromatographic separations are required in all cases.

The broader significance of the work lies in the provision of clearly navigable pathways toward the porpholactones that were selected by our own laboratory experience from among the many complementary and competing pathways described in the literature. While the porpholactones described are of broad utility, access to the free base porpholactones also enables entry to a number of other metalloporpholactones. Moreover, closely related porphyrins of utility can be derived, like the thionolactones used as hypochlorite sensors [18], the porpholactone reduction products porpholactols [4,55,56,57] useful as photosensitizers [58] or hydrogen evolution catalysts [28], as well as chlorolactones and chlorolactams [59,60], porpholactams [59,60], and imidazoloporphyrins [60], even if alternative synthetic pathways for the latter porphyrinoid were developed that circumvent the intermediate porpholactone [61]. The method described also allow direct access to the *meso*-pentafluorophenyl-substituted porphodilactone isomers of broad utility [17], though longer but more rational syntheses might be desirable when more than 10^−5^ mol quantities are desired of either the fluorinated or non-fluorinated series [17,62].

We hope this work will encourage more researchers outside the synthetic porphyrin community to prepare and study these intriguing porphyrinoids.

## Supplementary Materials

Figure S1: Image of 1 g ampule of OsO4, Figure S2: Image of H2S reaction set-up, Figure S3: 1H NMR (400 MHz, CDCl3) spectrum of *meso*-tetraphenyl-2,3-*cis*-dihydroxychlorin **2**, Figure S4: UV-vis spectrum (CH2Cl2) of *meso*-tetraphenyl-2,3-*cis*-dihydroxychlorin **2**, Figure S5: Image of the reaction flask, Figure S6: Image of the silica gel flash column chromatographic separation of the reaction mixture, Figure S7: 1H NMR (400 MHz, CDCl3) spectrum of TPL, Figure S8: UV-vis spectrum (CH2Cl2) of TPL, Figure S9: 1H NMR (400 MHz, CDCl3) spectrum of TPLZn, Figure S10: UV-vis spectrum (CH2Cl2) of TPLZn, Figure S11: 1H NMR (400 MHz, CDCl3) spectrum of TPLPt, Figure S12: UV-vis spectrum (CH2Cl2) of TPLPt, Figure S13: Reaction setup, Figure S14: Separation funnel setup, Figure S15: 1H NMR (400 MHz, CDCl3) spectrum of TFPL, Figure S16: 19F NMR (376 MHz, CDCl3) of TFPL, Figure S17: UV-vis spectrum (CH2Cl2) of TFPL, Figure S18: Representative TLC (CH2Cl2/25% hexanes) of the reaction mixture (RM) in comparison to the starting materials (SM) and product TFPLZn, Figure S19: 1H NMR (400 MHz, CDCl3) of TFPLZn, Figure S20: 19F NMR (376 MHz, CDCl3) of TFPLZn, Figure S21: UV-vis spectrum (CH2Cl2) of TFPLZn, Figure S22: 1H NMR (400 MHz, CDCl3) of TFPLPt, Figure S23: 19F NMR (376 MHz, CDCl3) of TFPLPt, Figure S24: UV-vis spectrum (CH2Cl2) of TFPLPt.

## Abbreviations

AcOH	acetic acid
AgOAc	silver acetate
bipy	2,2′-bipyridine
18-C-6	18-crown-6 crown ether
CTAP	cetyltrimethylammonium permanganate
DMF	dimethyl formamide
MCPBA	*m*-chloroperoxybenzoic acid
PhCN	benzonitrile
py	pyridine
