# Supplementary material for: Oxazolochlorins 21. Most Efficient Access to meso-Tetraphenyl- and meso-Tetrakis(pentafluorophenyl)porpholactones, and Their Zinc(II) and Platinum(II) Complexes
Source: Molecules. 2020 Sep 22;25(18):4351. doi: 10.3390/molecules25184351 (PMC7570530; doi:10.3390/molecules25184351)
Supplement: Supplementary file 1 [file molecules-25-04351-s001.pdf]

Electronic Supporting Information (ESI) to:

Oxazolochlorins 21. Most Efficient Access to *meso*-Tetraphenyl- and *meso*-Tetrakis(pentafluorophenyl)-porpholactones, and their Zinc(II) and Platinum(II) Complexes

*Damaris Thuita, Dinusha Damunupola, and Christian Brückner**

Department of Chemistry
University of Connecticut, Unit 3060
Storrs, CT 06269-3060
Unites States

* Corresponding author: c.bruckner@uconn.edu

Table of Contents

MATERIALS AND INSTRUMENTS **S3**

TWO-STEP PROCEDURE FOR SYNTHESIS OF *MESO*-TETRAPHENYL-2-OXA-3-OXOPORPHYRIN (TPL) BY OsO_4-MEDIATED DIHYDROXYLATION, FOLLOWED BY CrO_3 OXIDATION **S4**

Step 1: Dihydroxylation of *meso*-Tetraphenylporphyrin: *meso*-Tetraphenyl-2,3-*cis*-dihydroxychlorin 2 .... **S4**

Figure S1. Image of 1 g ampule of OsO_4 .... S6

Figure S2. Image of H_2S reaction set-up. S6

Figure S3. ^{1}H NMR (400 MHz, $CDCl_3$) spectrum of *meso*-tetraphenyl-2,3-*cis*-dihydroxychlorin **2**. S6

Figure S4. UV-vis spectrum (CH_2Cl_2) of *meso*-tetraphenyl-2,3-*cis*-dihydroxychlorin **2**. S7

Step 2: Synthesis of *meso*-Tetraphenyl-2-oxa-3-oxoporphyrin (TPL) via CrO_3 Oxidation **S7**

Figure S5. Image of the reaction flask. S9

Figure S6. Image of the silica gel flash column chromatographic separation of the reaction mixture. S9

Figure S7. ^{1}H NMR (400 MHz, $CDCl_3$) spectrum of TPL. S10

Figure S8. UV-vis spectrum (CH_2Cl_2) of TPL. S10

[*meso*-Tetraphenyl-2-oxa-3-oxoporphyrinato]zinc(II) (TPLZn) S11

Figure S9. ^{1}H NMR (400 MHz, $CDCl_3$) spectrum of TPLZn. S12

Figure S10. UV-vis spectrum (CH_2Cl_2) of TPLZn. S12

Synthesis of [*meso*-Tetraphenyl-2-oxa-3-oxoporphyrinato]Pt(II) (TPLPt) S13

Figure S11. ^{1}H NMR (400 MHz, $CDCl_3$) spectrum of TPLPt. S14

Figure S12. UV-vis spectrum (CH_2Cl_2) of TPLPt. S14

ONE-STEP SYNTHESIS OF *MESO*-TETRAKIS(PENTAFLUOROPHENYL)-2-OXA-3-OXOPORPHYRIN (T^FPL) BY $RuCl_3$ / OXONE® MEDIATED OXIDATION. GENERAL PROCEDURE. S15

Figure S13. Reaction setup. S16

Figure S14. Separation funnel setup. S16

Figure S15. ^{1}H NMR (400 MHz, $CDCl_3$) spectrum of T^FPL. S17

Figure S16. ^{19}F NMR (376 MHz, $CDCl_3$) of T^FPL. S17

Figure S17. UV-vis spectrum (CH_2Cl_2) of T^FPL. S18

Synthesis of [*meso*-Tetrakis(pentafluorophenyl)-2-oxa-3-oxoporphyrinato]zinc(II) (T^FPLZn) S19

Figure S18. Representative TLC (CH_2Cl_2/25% hexanes) of the reaction mixture (RM) in comparison to the starting materials (SM) and product T^FPLZn. S20

Figure S19. ^{1}H NMR (400 MHz, $CDCl_3$) of T^FPLZn. S20

Figure S20. ^{19}F NMR (376 MHz, $CDCl_3$) of T^FPLZn. S20

Figure S21. UV-vis spectrum (CH_2Cl_2) of T^FPLZn. S21

Synthesis of [*meso*-Tetrakis(pentafluorophenyl)-2-oxa-3-oxoporphyrinato]platinum(II) (T^FPLPt) S22

Figure S22. ^{1}H NMR (400 MHz, $CDCl_3$) of T^FPLPt. S23

Figure S23. ^{19}F NMR (376 MHz, $CDCl_3$) of T^FPLPt. S23

Figure S24. UV-vis spectrum (CH_2Cl_2) of T^FPLPt. S23

ESI REFERENCES S24

MATERIALS AND INSTRUMENTS

All solvents and reagents (Sigma-Aldrich, St. Louis, MO and Acros, Fair Lawn NJ) were used as received. $T^{F}PP$ [1] was either synthesized according to the literature procedure or obtained commercially [2]; $T^{F}PPZn$ [1] and $T^{F}PPPt$ [3] were synthesized by metal insertion into the free base according to literature procedures. TPP was synthesized according to a literature procedure [4]; is also commercially readily available.

All solvents were reagent-grade, or better, and were used as received. The $CHCl_3$ used was EtOH-stabilized (amylene-stabilized $CHCl_3$, for example, is unsuitable for the osmylations!). CTAP was prepared as described in the literature [5].

Oxone® is an oxidizing agent; a mixture of the composition of $2KHSO_5 \cdot KHSO_4 \cdot K_2SO_4$.

Aluminum-backed, silica gel 60, 250 µm thickness analytical plates were used for analytical TLC; Either 20 × 20 cm, glass-backed, silica gel 60, 500 µm thickness preparative TLC plates or standard grade, 60 Å, 32-63 µm flash column silica gel were used for the preparative chromatography.

^{1}H and ^{19}F NMR spectra were recorded on a Bruker 300, 400 or 500 MHz instruments in the solvents indicated, and were referenced to residual solvent peaks or internal TMS. All UV-vis spectra were recorded on Cary 50 or 100 UV-vis spectrometers (Varian).

TWO-STEP PROCEDURE FOR SYNTHESIS OF *MESO*-TETRAPHENYL-2-OXA-3-OXOPORPHYRIN (TPL) BY OsO_4-MEDIATED DIHYDROXYLATION, FOLLOWED BY CrO_3 OXIDATION.

Step 1: Dihydroxylation of *meso*-Tetraphenylporphyrin: *meso*-Tetraphenyl-2,3-*cis*-dihydroxychlorin 2.

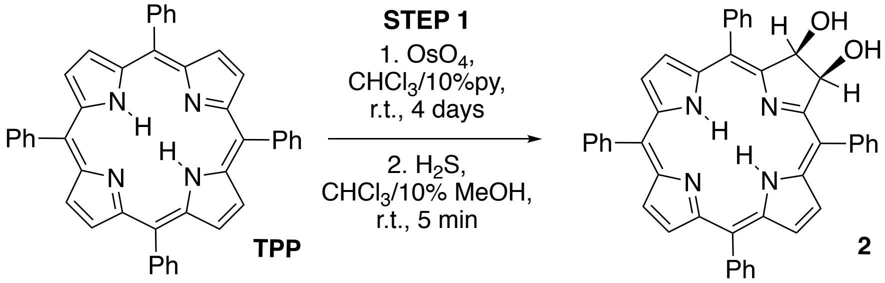

Prepared according to a literature procedure (Note 1) [6]. To a 500 mL round-bottom flask equipped with a stir bar, *meso*-tetraphenylporphyrin (TPP) (2.20 g, 3.58 mmol) was dissolved in a mixture of $CHCl_3$ (270 ml, EtOH-stabilized) and freshly distilled pyridine (30 mL). A glass ampoule of 1.0 g of OsO_4 (1.0 g, 3.93 mmol, ~1.1 equiv.) was opened (Note 2) and immediately submerged (cap and body) (Figure S1) into the mixture. The flask was then stoppered, shielded from light with aluminum foil, and stirred at ambient temperature (20°C– 24°C) The progress of the reaction was monitored by occasional TLC. Once no further progress was noted (after about 4 days), the solvent was removed on a rotary evaporator as best as possible. The crude osmate ester product was then dissolved in a solution of 10% $MeOH/CHCl_3$ (~250 mL). The osmate ester was then reductively cleaved by purging with gaseous H_2S for 5 min (Note 2); the mixture was stirred for an additional 30 min before the majority of the excess H_2S was purged from the solution by a stream of nitrogen gas through the solution (Note 3). Following the addition of further MeOH (20 mL), the precipitated black OsS was filtered off through a short plug of Celite®. The filtrate was evaporated to dryness by a stream of N_2 or rotary evaporation (Note 4). The resulting residue was dissolved in a minimal amount of $CHCl_3$ and loaded onto a silica gel column (25 × 7 cm) and eluted with CH_2Cl_2. The first fraction was starting material TPP (250 mg, 11% recovery). CH_2Cl_2/1.5% MeOH then eluted diol chlorin **2**. Slow evaporation from a $MeOH/CH_2Cl_2$ mixture provided product **2** as a bright purple crystalline material (1.00 g, 1.40 mmol, 49% yield). R_f (silica-CH_2Cl_2/1.5% MeOH) = 0.68. ^1H NMR (400 MHz, $CDCl_3$, δ):

8.67 (d, ^3J = 4.9 Hz, 1H), 8.51 (s, 1H), 8.36 (d, ^3J = 4.9 Hz, 1H), 8.19 (br d, ^3J = 6.4 Hz, 2H), 8.12 (d, ^3J = 6.2 Hz, 1H), 7.97 (d, ^3J = 6.1 Hz, 1H), 7.79– 7.73 (m, 6H), 6.40 (s, 1H), 3.15 (s, exchangeable with D_2O, 1H), –1.75 (s, 1H). ^{13}C NMR (125 MHz, $CDCl_3$, δ) [6]: 73.9, 113.2, 123.1, 124.2, 126.7, 127.5, 127.7, 127.9, 128.1, 132.2, 132.7, 133.9, 134.1, 135.5, 140.6, 141.2, 141.8, 153.2, 161.4 ppm. UV-vis (CH_2Cl_2) λ_{max} (log ε) 408 (5.27), 518 (4.19), 544 (4.19), 592 (3.85), 644 (4.38) nm. HR-MS (ESI+, 100% CH_3CN, 30 V cone voltage) [6]: *m/z* = 648.2525 calc'd for $C_{44}H_{32}N_4O_2$ $[M]^+$; found 648.2525.

Notes

1. All the steps except for the solvent removal were performed in a fume hood following usual laboratory safety practices (gloves, eye protection and lab coats).
2. CAUTION: Note the hazard and risk of using OsO_4 and H_2S. Trap all excess H_2S (bleach traps, for example).
3. CAUTION: Work in a fume hood and trap all purged H_2S (e.g., bleach bubbler)! NB: Washing the organic reaction mixture with dilute base solutions to extract the H_2S tends to form stable emulsions and thus is not a viable alternative for the removal of the excess H_2S. Alternatively, an aqueous solution of $NaHSO_3$ can be used to reductively cleave the osmate ester [11], but the reaction is slower and lower yielding, and best suited for the *meso*-pentafluorophenyl derivatives that cannot be treated with H_2S (the sulfides undergo nucleophilic aromatic substitution reactions with the *p*-fluorine atoms).
4. CAUTION: Work in a fume hood to capture all residual H_2S!

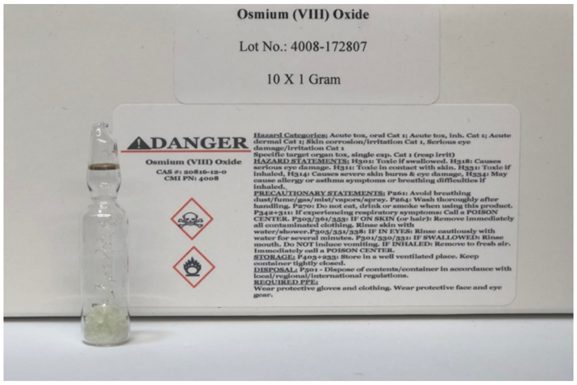

Figure S1. Image of 1 g ampule of OsO_4.

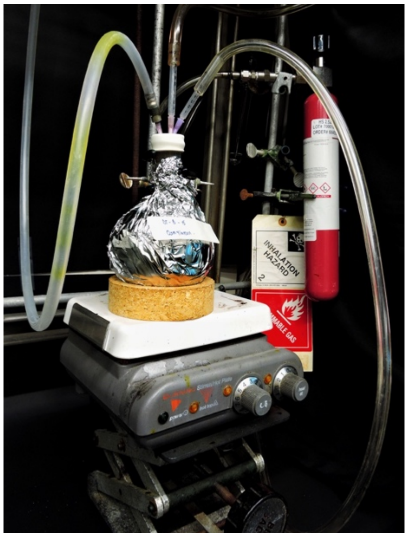

Figure S2. Image of H_2S reaction set-up.

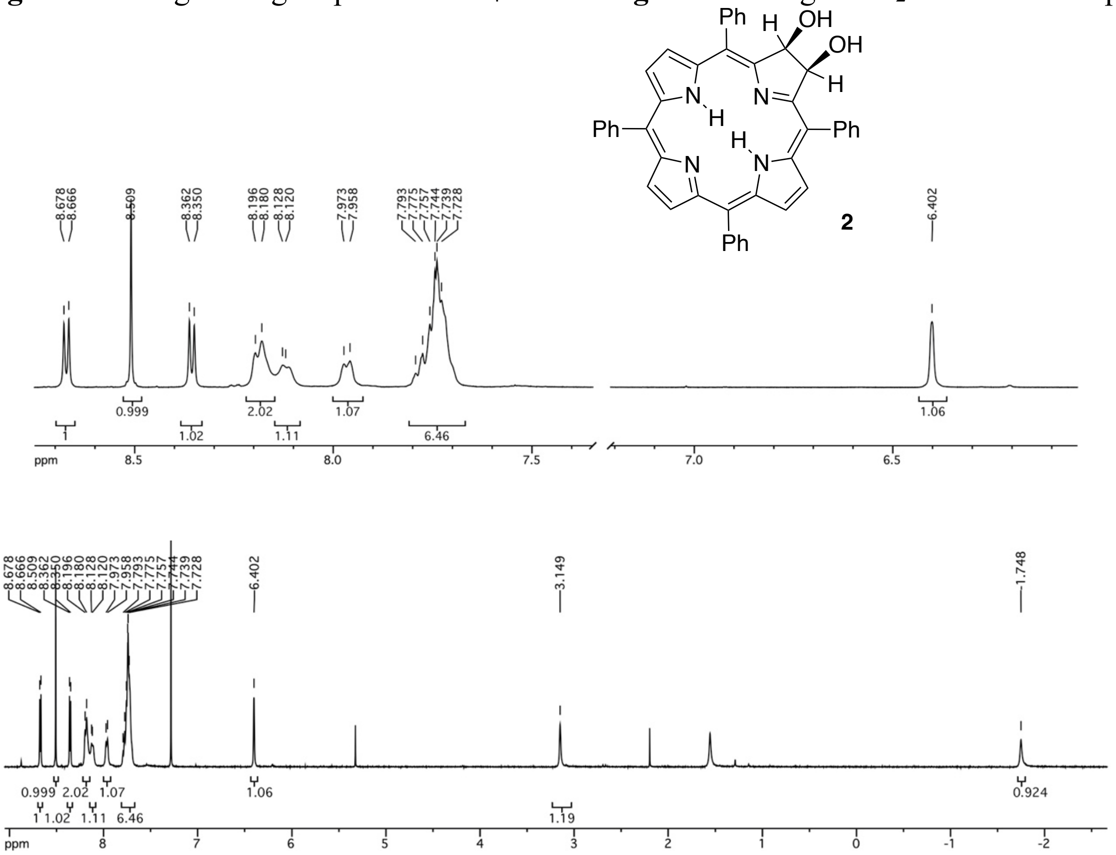

Figure S3. ^{1}H NMR (400 MHz, $CDCl_3$) spectrum of *meso*-tetraphenyl-2,3-*cis*-dihydroxychlorin **2**.

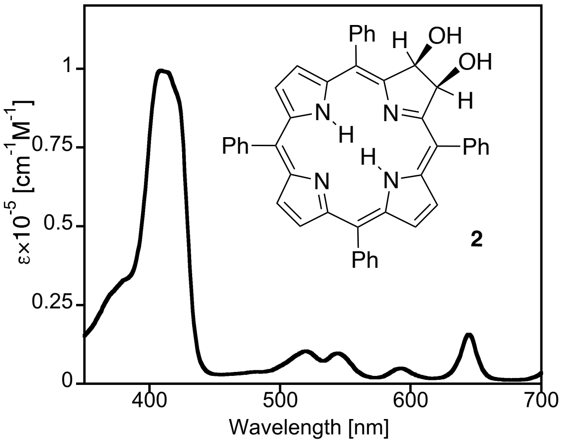

Figure S4. UV-vis spectrum (CH_2Cl_2) of *meso*-tetraphenyl-2,3-*cis*-dihydroxychlorin **2**.

Step 2: Synthesis of *meso*-Tetraphenyl-2-oxa-3-oxoporphyrin (TPL) via CrO_3 Oxidation of Chlorin Diol 2

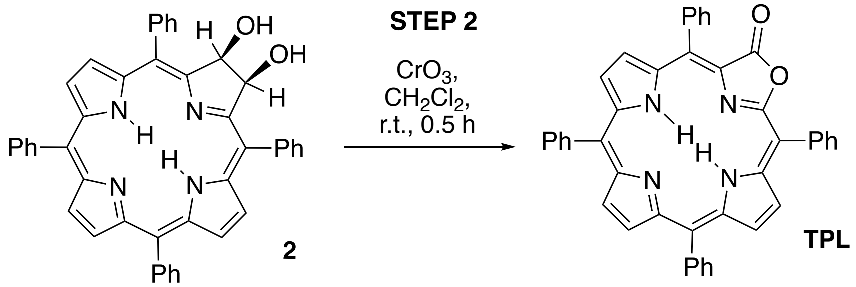

Prepared according to a literature procedure (Note 1) [8]. In a 25 mL round-bottom flask equipped with a stir bar and two carbon pellets, *meso*-tetraphenyl-2,3-*cis*-dihydroxychlorin **2** (100 mg, 0.154 mmol) was dissolved in pyridine (5 mL). Chromium trioxide CrO_3 (~10 equiv., 154 mg, 1.54 mmol) was added to the solution. The round-bottom flask was shielded from light with aluminum foil, and stirred at ambient temperature (Figure S5). The disappearance of the starting material and the appearance of the product were monitored by TLC. Once no further progress of the reaction was detectable (after ~30 min), CH_2Cl_2 (25 mL) was added and the mixture was transferred into a 125 mL separatory funnel and washed with water (3 × 25 ml). The organic phase was separated and filtered through a short plug of diatomaceous earth (Celite®)

and dried over anhyd. Na_2SO_4. The drying agent was removed by gravity filtration and the filtrate was evaporated to dryness by rotary evaporation. A gentle stream of N_2 passed through the crude material for several h ensured that the crude material was thoroughly dried. The crude material was dissolved in a few mL CH_2Cl_2 coated onto silica. The material was then dry-loaded onto a flash column (~6 × 25 cm) silica–CH_2Cl_2) and chromatographed to provide TPL in 40–60% yield (60 mg) as a pinkish red solid (Figure S6). R_f (silica–CH_2Cl_2) = 0.67. ^{1}H NMR (400 MHz, $CDCl_3$, δ): 8.82 (dd, ^3J = 4.8, ^4J = 1.8 Hz, 1H), 8.79 (dd, ^3J = 4.8, ^4J = 1.8 Hz, 1H), 8.73 (dd, ^3J = 4.8, ^4J = 1.8 Hz, 1H), 8.62 (d, ^3J = 4.4 Hz, 1H), 8.60 (dd, ^3J = 4.4, ^4J = 1.8 Hz, 1H), 8.56 (d, ^3J = 4.6 Hz, 1H), 8.17–8.12 (m, 6H), 8.02–7.99 (m, 2H), 7.80–7.74 (m, 12H), –1.62 (s, 1H), –2.00 (s, 1H). ^{13}C NMR (100 MHz, $CDCl_3$, δ) [7]: 170.2, 155.2, 154.9, 154.7, 141.4, 140.8, 137.6, 137.4, 137.0, 136.7, 135.4, 134.7, 134.6, 132.9, 131.8, 131.1, 129.9, 129.0, 128.4, 128.3, 128.25, 127.5, 127.3, 126.35, 124.3, 107.0, 102.6, 101.1 ppm. UV-vis (CH_2Cl_2) λ_{max} (log ε): 409 (5.23), 511 (3.92), 549 (4.00), 581 (3.76), 633 (3.81) nm. HR-MS (FAB, 3-NBA, quadrupole) [7]: *m/e* calc'd for $C_{43}H_{28}N_4O_2$: 632.2212, found 632.2148. HR-MS (ESI+, 100% CH_3CN, 30 V cone voltage) [7]: *m/z* = 633.2291 calc'd for $C_{43}H_{29}N_4O_2$ $[M+H]^+$; found 633.2309.

Note: All the steps except for the solvent removal were performed in a fume hood following usual laboratory safety practices (gloves, eye protection and lab coats).

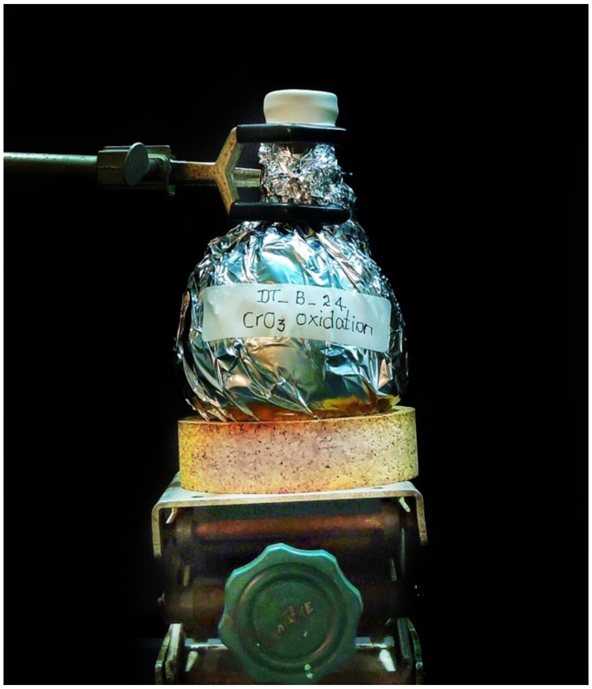

Figure S5. Image of the reaction flask.

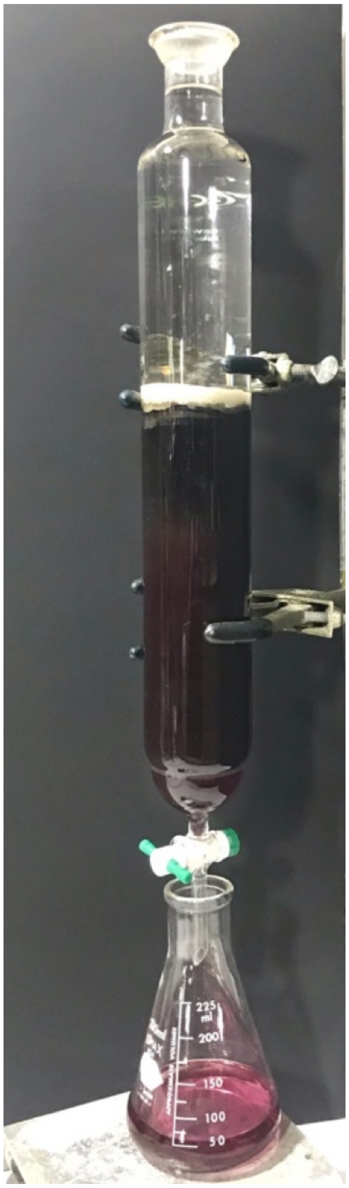

Figure S6. Image of the silica gel flash column chromatographic separation of the reaction mixture.

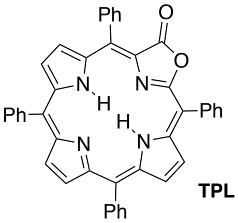

TPL

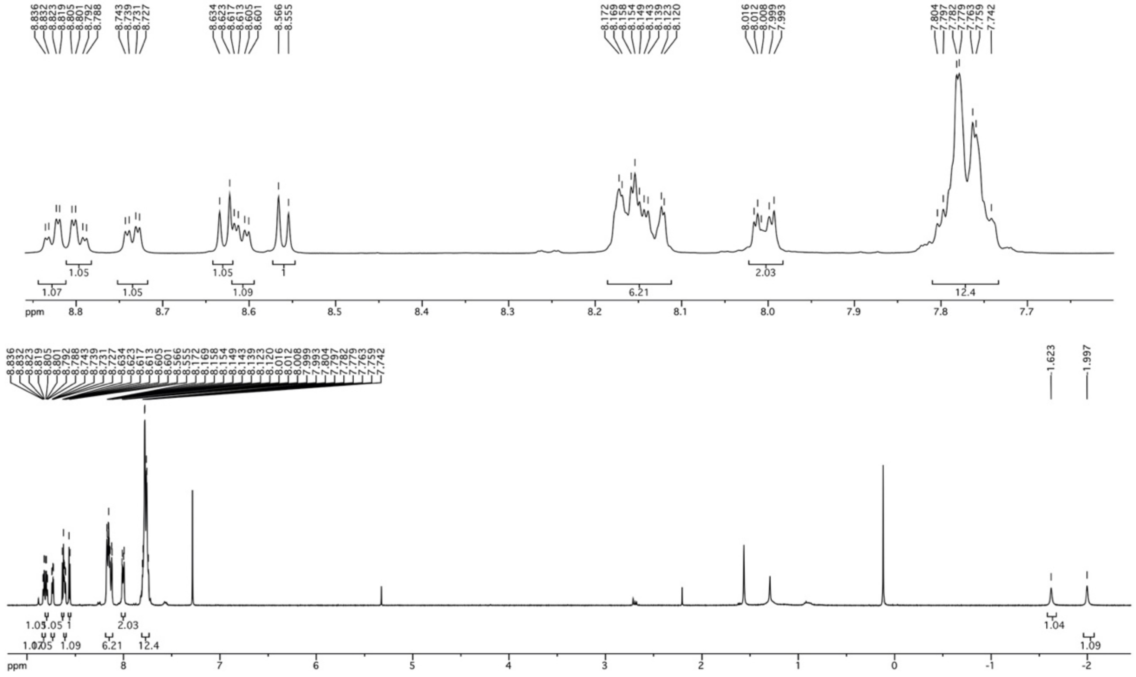

Figure S7. ^{1}H NMR (400 MHz, $CDCl_3$) spectrum of TPL.

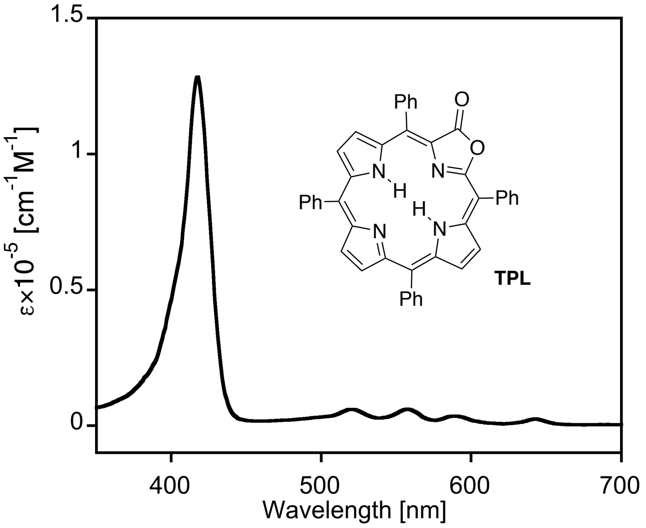

Figure S8. UV-vis spectrum (CH_2Cl_2) of TPL.

PREPARATION OF METALLOPORPHOLACTONE TPLZN AND TPLPT VIA METAL INSERTION.

[*meso*-Tetraphenyl-2-oxa-3-oxoporphyrinato]zinc(II) (TPLZn)

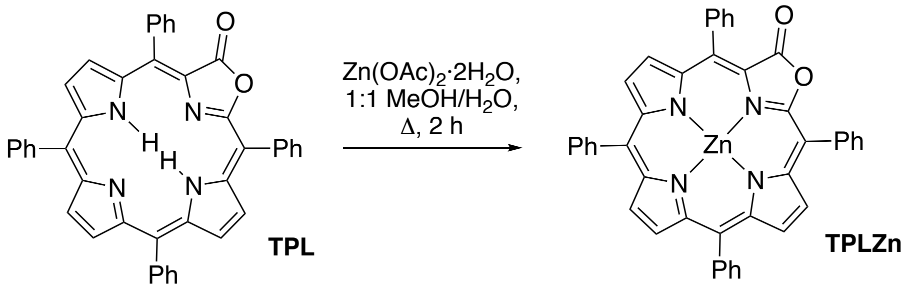

Prepared as reported in the literature [7]. A 250 ml round-bottom flask was equipped with a stir bar, heating mantle, and a reflux condenser. The flask was charged with porpholactone TPL (123 mg, 1.94 mmol) and $Zn(OAc)_2{\cdot}2H_2O$ (123 mg, 0.193 mmol, 3 equiv.) dissolved in $CHCl_3$/MeOH (8:1, 80 mL). The reaction mixture was heated to reflux for ~2 h. Upon completion of reaction as observed by TLC, the reaction mixture was cooled to room temperature and filtered through Celite®. The filtrate was concentrated, the concentrate was loaded onto the silica gel column, and purified by short column chromatography (silica, CH_2Cl_2) to produce TPLZn as a purple-colored film. Recrystallization by dissolution in a few mL of CH_2Cl_2, addition of ~50% MeOH, and slow removal of the CH_2Cl_2 on a rotary evaporator (at ambient T) precipitated TPLZn; it was retrieved by filtration and air-dried to provide TPLZn as a bright purple crystalline solid in 97% yield (130 mg, 0.143 mmol). R_f (silica–CH_2Cl_2) = 0.44. ^{1}H NMR (400 MHz, $CDCl_3$, *δ*): 8.79 (d, ^3J = 4.8 Hz, 1H), 8.74–8.71 (m, 3H), 8.64 (d, ^3J = 4.6 Hz, 1H), 8.55 (d, ^3J = 4.6 Hz, 1H), 8.13 (m, 4H), 8.09 (dd, ^3J = 7.7, ^4J = 1.6 Hz, 2H), 7.97 (dd, ^3J = 7.5, ^4J = 1.6 Hz, 2H), 7.79–7.72 (m, 12H). ^{13}C NMR (75 MHz, $CDCl_3$, δ) [7]: 173.5, 154.1, 143.4, 142.1, 142.0, 139.0, 138.0, 134.3, 134.0, 133.8, 132.4, 132.3, 132.2, 132.0, 130.9, 130.7, 130.6, 129.5, 128.8, 127.9, 127.8, 127.7, 127.6, 127.5, 126.7, 126.6. UV-vis (CH_2Cl_2) λ_{max} (log ε): 402 (sh), 422 (5.53), 520 (3.54), 558 (4.07), 602 (4.44) nm. HR-MS (DART+, orifice voltage = 30 V, 100% CH_3CN, TOF) [7]: *m*/*z* = 695.1425 calc'd for $C_{43}H_{27}N_4O_2{}^{64}Zn$ $[MH]^+$; found 695.1429.

Note: All the steps except for the solvent removal were performed in a fume hood following usual laboratory safety practices (gloves, eye protection and lab coats).

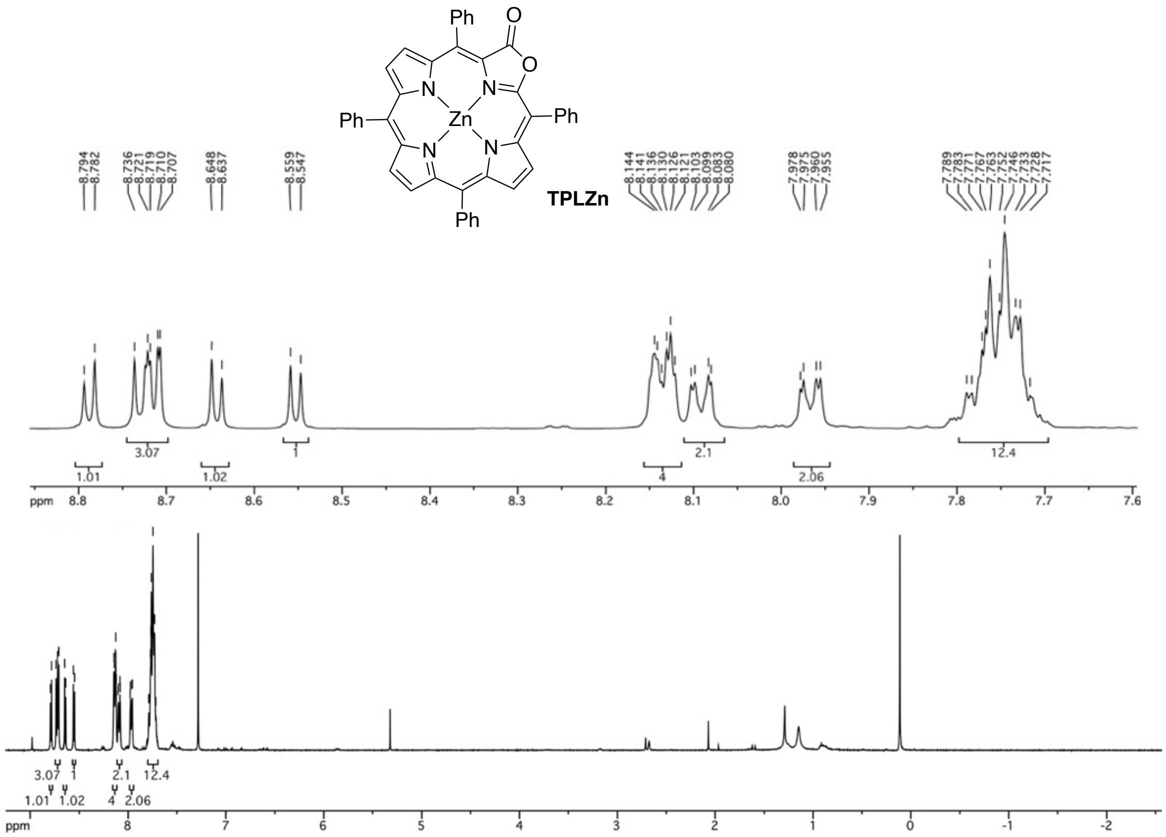

Figure S9. ^{1}H NMR (400 MHz, $CDCl_3$) spectrum of **TPLZn**.

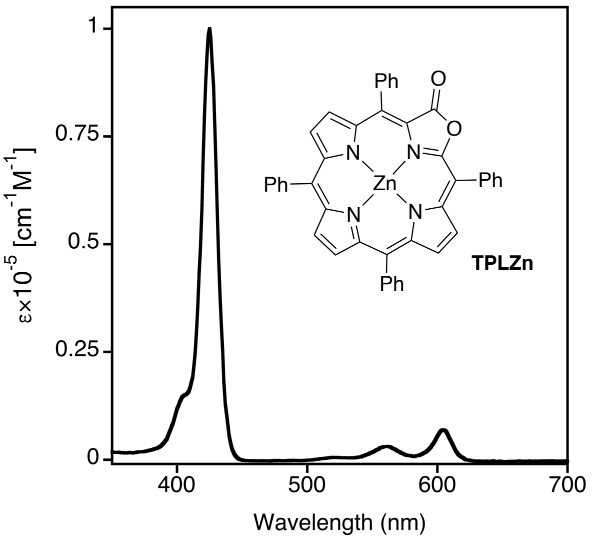

Figure S10. UV-vis spectrum (CH_2Cl_2) of **TPLZn**.

Synthesis of [*meso*-Tetraphenyl-2-oxa-3-oxoporphyrinato]Pt(II) (TPLPt).

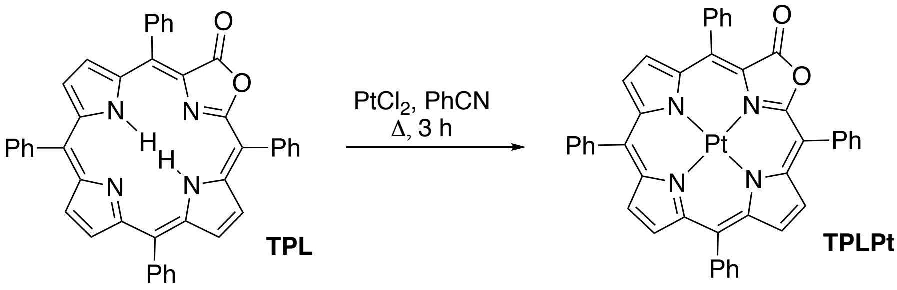

Prepared as reported in the literature [7]. A 50 ml round-bottom flask was equipped with a stir bar, heating mantle, a reflux condenser and a N_2 inlet. TPL (65.0 mg, 0.086 mmol) was dissolved in benzonitrile PhCN (5 mL) and added to a refluxing solution of benzonitrile PhCN (20 mL) and $PtCl_2$ (61 mg, 0.344 mmol, 4 equiv.). The mixture was heated to reflux for 3 h. When the starting material was consumed (reaction monitored by TLC), the reaction mixture was allowed to cool and was evaporated to dryness by rotary evaporation. The resulting mixture was dissolved in CH_2Cl_2 (2–5ml), loaded as solution onto a flash column (silica-CH_2Cl_2) and chromatographed. TPLPt was isolated in 71% (50 mg) yield as a magenta powder. R_f (silica-CH_2Cl_2) = 0.17. ^{1}H NMR (400 MHz, $CDCl_3$, δ): 8.67 (d, ^3J = 5.0 Hz, 1H), 8.65–8.62 (m, 4H), 8.51 (d, ^3J = 5.0 Hz, 1H), 8.11–8.05 (m, 6H), 7.96 (dd, ^3J = 7.6, ^4J = 1.6 Hz, 2H), 7.79–7.72 (m, 12H). ^{13}C NMR (100 MHz, $CDCl_3$, δ) [10]: 164.7, 148.2, 145.8, 142.9, 140.9, 140.5, 140.4, 139.0, 138.9, 138.3, 137.6, 136.5, 133.6 (2 overlapping signals), 133.4, 133.3, 132.1, 131.1, 130.1, 129.6, 129.3, 128.7, 128.4, 128.3 (2 overlapping signals), 128.2, 128.0, 127.8, 127.5, 127.1 (2 overlapping signals), 124.1, 122.1, 116.6, 102.9 ppm. UV–vis (CH_2Cl_2) λ_{max} (log ε): 421 (5.14), 527 (4.07), 568 (4.49) nm. HR-MS (ESI+, orifice voltage = 30 V, 100% CH_3CN, TOF) [7]: m/z = 826.1782 calc'd for $C_{43}H_{27}N_4O_2Pt$ $[MH]^+$; found 826.1802.

Note: All the steps except for the solvent removal were performed in a fume hood following usual laboratory safety practices (gloves, eye protection and lab coats).

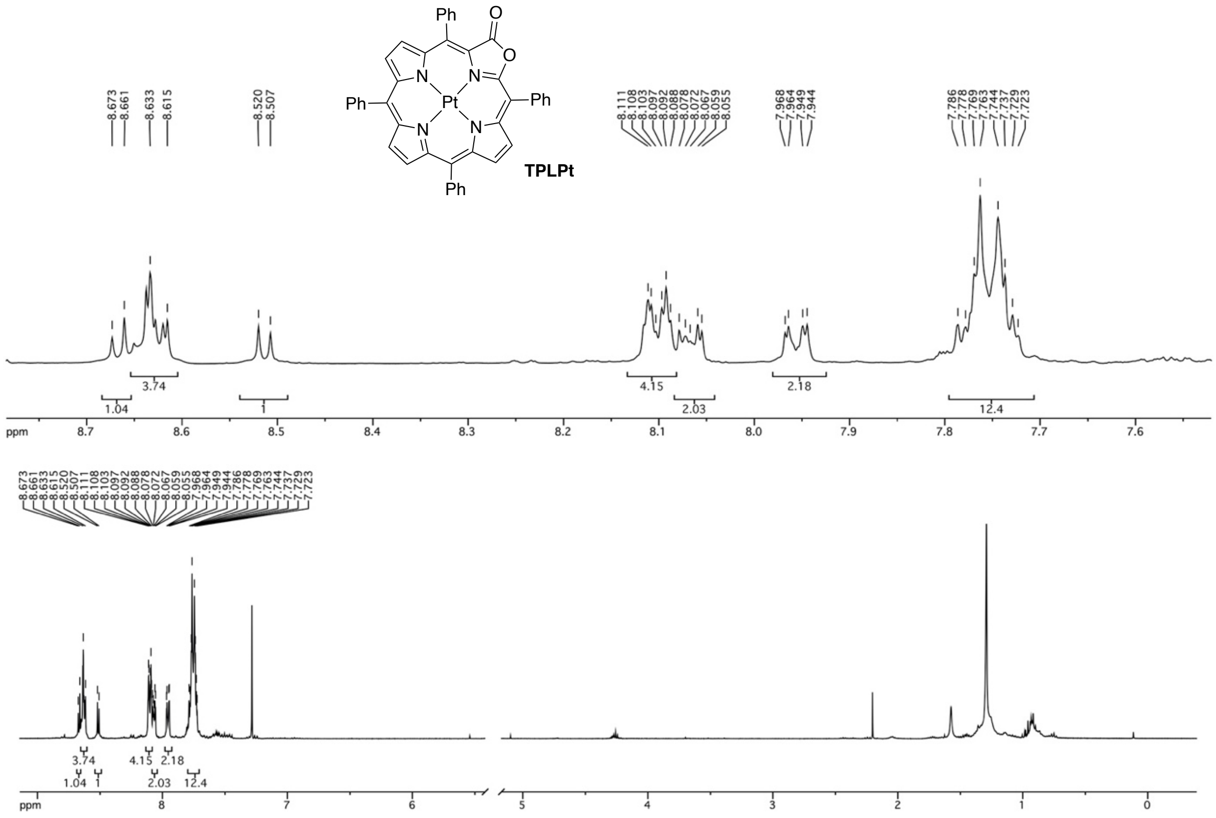

Figure S11. ^{1}H NMR (400 MHz, $CDCl_3$) spectrum of **TPLPt**.

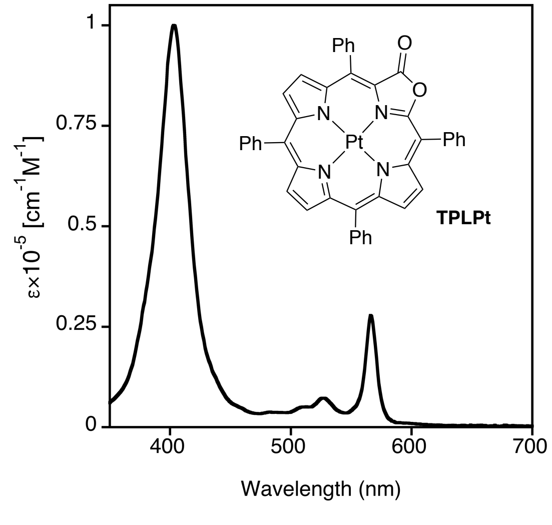

Figure S12. UV-vis spectrum (CH_2Cl_2) of **TPLPt**.

ONE-STEP SYNTHESIS OF *MESO*-TETRAKIS(PENTAFLUOROPHENYL)-2-OXA-3-OXOPORPHYRIN T^{F}PL BY $RuCl_3$ / OXONE® MEDIATED OXIDATION. GENERAL PROCEDURE.

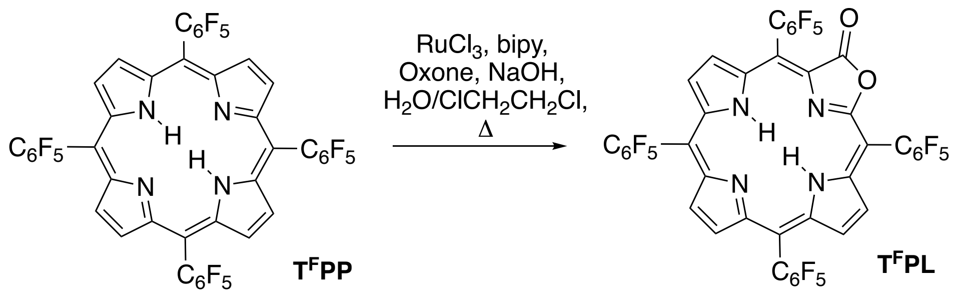

Prepared according to a modified procedure reported in literature (Note 1) [9]. In a 750 ml round-bottom (or a two neck round-bottom) flask equipped with a stir bar and reflux condenser, T^{F}PP (0.8 g, 0.8 mmol, 1 equiv.) and $RuCl_3$ (85 mg, 0.4 mmol, 0.5 equiv.) were dissolved in 1,2-dichloroethane ($ClCH_2CH_2Cl$, 250 mL) (Note 2). A solution containing 2,2'-bipyridine (65 mg, 0.4 mmol, 0.5 equiv.) in $ClCH_2CH_2Cl$ (7 mL) and water (250 mL) were added. A condenser was attached to one of the necks and the solution was heated to reflux (Figure S13). A mixture of Oxone® (7.4 g, 12 mmol, 15 equiv.) and NaOH (480 mg, 12 mmol, 15 equiv.) in water (15 mL) was added periodically in five equal portions over the course of 5 h to the refluxing solution. After completion of the addition, the reaction mixture was refluxed overnight. The mixture was allowed to cool to room temperature, quenched with a saturated aqueous solution of $Na_2S_2O_3$ (2.0 g in 5 mL of water). The reaction mixture was stirred, allowed to stand for 30 min, and transferred to a 750 mL separatory funnel (Figure S14). The (bottom) organic layer was separated, and the aqueous layer was extracted twice using CH_2Cl_2 (2 × 150 mL). The combined organic fractions were washed with a saturated aqueous NaCl solution (2 × 150 mL). The organic layer was dried with anhydrous Na_2SO_4 (30 min) and the drying agent was removed by gravity filtering. The filtrate was reduced to dryness using rotary evaporation (in a 500 mL round-bottom flask). The residue was dissolved in CH_2Cl_2 (2–5ml), loaded as a solution onto a packed column (silica, 5 × 60 cm, with a fritted disk, eluent: CH_2Cl_2/hexanes = 2:1) and separated to obtain the product T^{F}PL as a purple solid upon solvent removal in 30% yield (240 mg, 0.24 mmol). R_f (silica–CH_2Cl_2/20% hexane) = 0.68. ^{1}H NMR (400 MHz, $CDCl_3$, δ):

8.95 (d, ^{3}J = 5.4 Hz, 1H), 8.91–8.87 (two overlapping d, 2H), 8.85 (d, ^{3}J = 5.4 Hz, 1H), 8.69 (d, ^{3}J = 4.7 Hz, 1H), 8.62 (d, ^{3}J = 4.7 Hz, 1H), –1.76 (s, 1H), –2.06 (s, 1H). ^{13}C NMR (100 MHz, $CDCl_3$, δ) [11]: 165.8, 157.1, 154.7, 154.5, 141.2, 138.8, 138.5, 136.4, 135.3, 133.8, 131.8, 130.0, 127.9, 127.8, 126.0, 108.9, 105.3, 102.6, 87.4 ppm; all *meso*-aryl carbon atoms are broadened, often beyond distinction, because of F-C couplings. ^{19}F NMR (376 MHz, $CDCl_3$, δ): –136.70 (m, 4F), –137.03 (dd, ^{3}J = 22.7, ^{4}J = 7.1 Hz, 2F), –138.49 (dd, ^{3}J = 23.0, ^{4}J = 7.4 Hz, 2F), –150.38 to –150.58 (m, 3F), –151.20 (t, ^{3}J = 21.0 Hz, 1F), –160.78 (m, 6F), –161.42 (td, ^{3}J = 22.0, ^{4}J = 6.8 Hz, 2F). UV-vis (CH_2Cl_2) λ_{max} (log ε): 409 (5.18), 510 (3.95), 545 (3.81), 589 (3.60), 642 (4.03) nm. HR-MS (ESI+, 100% CH_3CN, TOF) [9]: *m/z* = 993.0402 calc'd. for $C_{43}H_9F_{20}N_4O_2$ $[MH]^+$; found 993.0400.

Notes:

1. All the steps except for the solvent removal were performed in a fume hood following usual laboratory safety practices (gloves, eye protection and lab coats).
2. The organic solvent used in biphasic reaction mixture, 1,2-dichloroethane is highly toxic and considered as a potential carcinogen. Protective eye wear, gloves, lab coats and masks are required when handling.

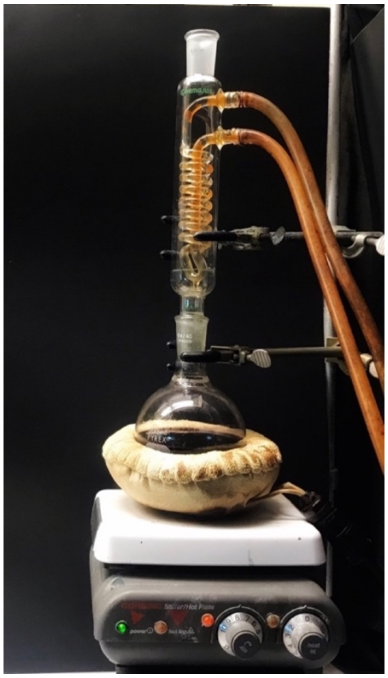

Figure S13. Reaction setup.

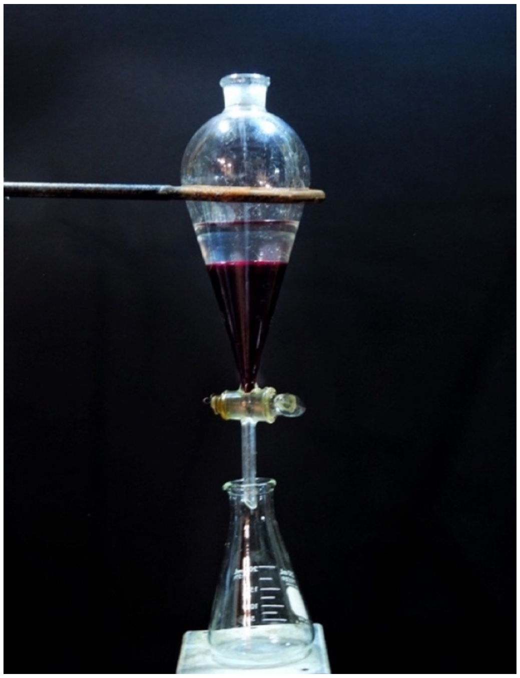

Figure S14. Separation funnel setup.

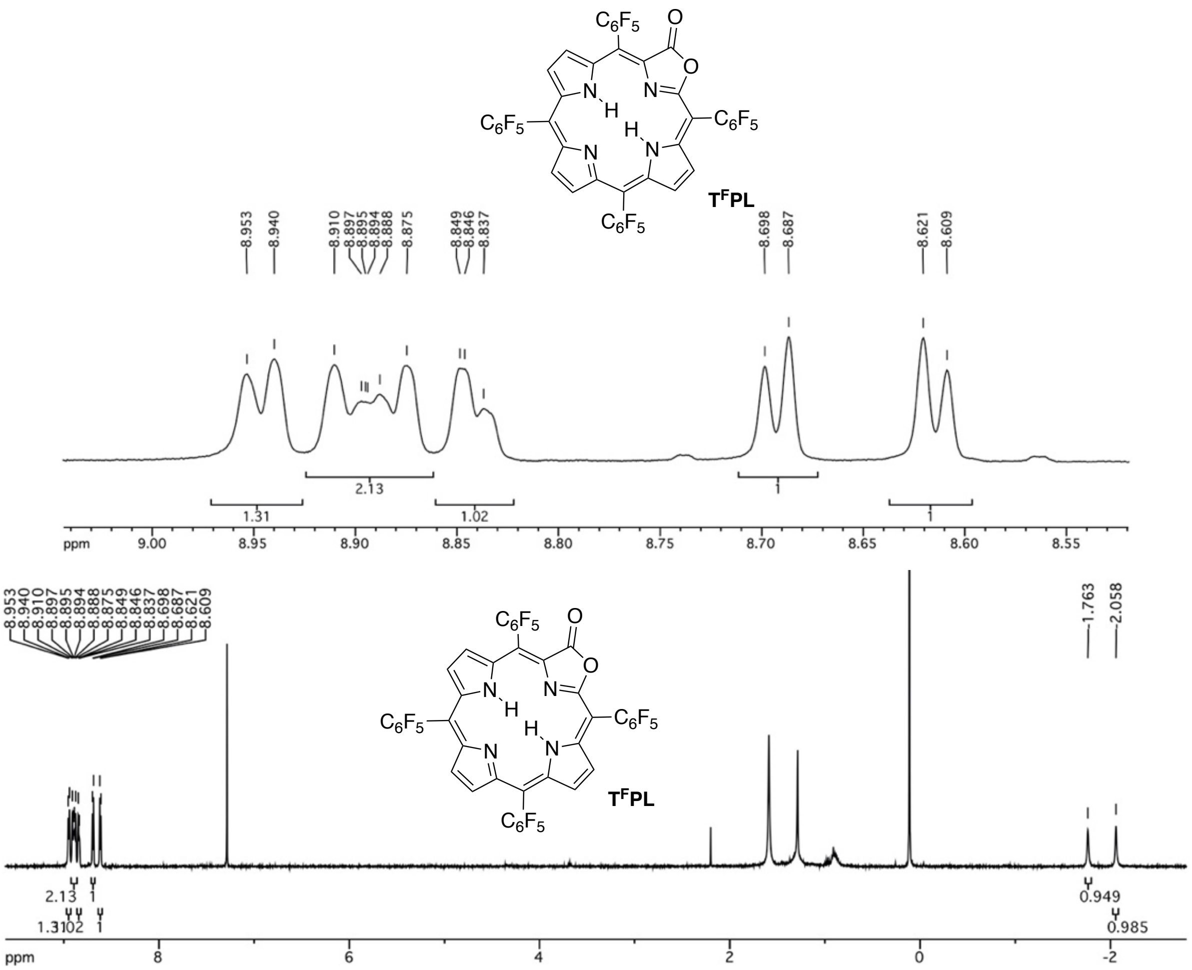

Figure S15. ^{1}H NMR (400 MHz, $CDCl_3$) spectrum of T^{F}PL.

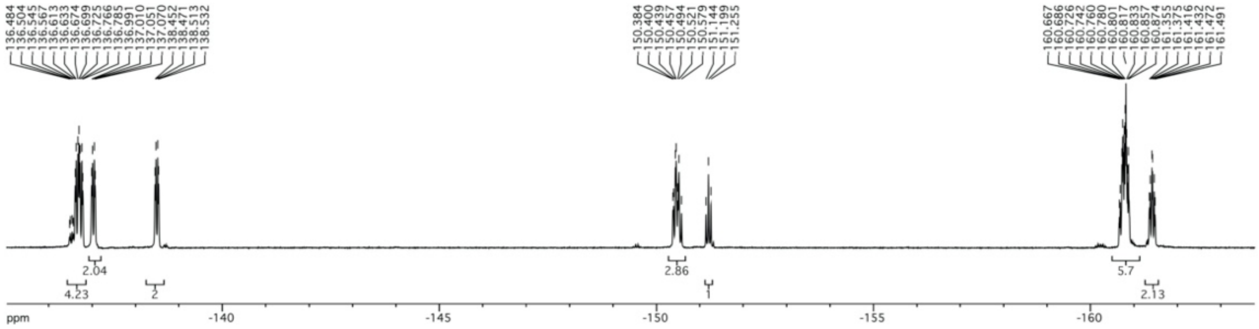

Figure S16. ^{19}F NMR (376 MHz, $CDCl_3$) of T^{F}PL.

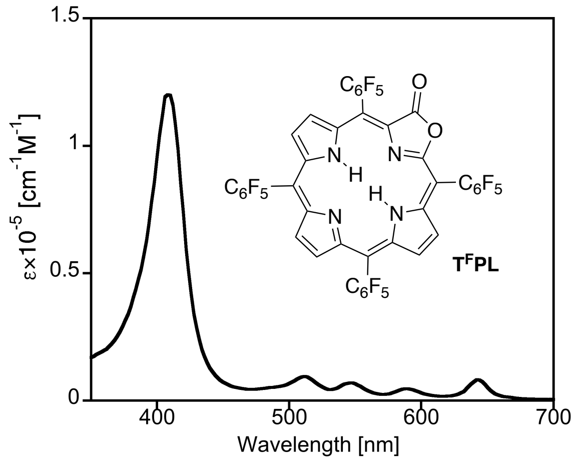

Figure S17. UV-vis spectrum (CH_2Cl_2) of T^{F}PL.

Synthesis of [*meso*-Tetrakis(pentafluorophenyl)-2-oxa-3-oxoporphyrinato]zinc(II) (T^{F}PLZn).

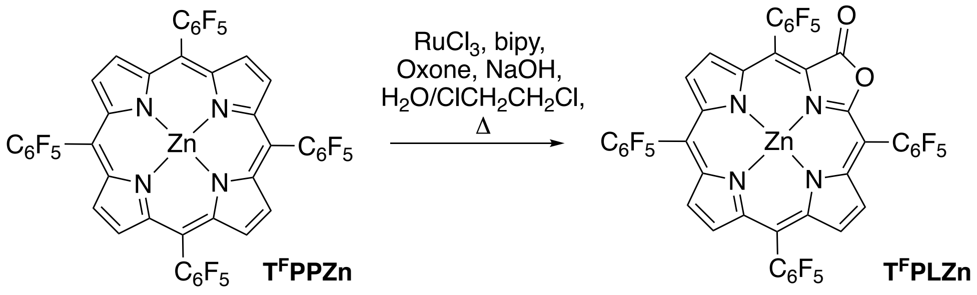

Prepared in 35% yield (189 mg) as a bright purple powder according to the general procedure for the synthesis of T^{F}PL synthesis using T^{F}PPZn (0.52 g, 0.5 mmol, 1 equiv.) and $RuCl_3$ (53 mg, 0.25 mmol, 0.5 equiv.) in $ClCH_2CH_2Cl$ (150 mL) and water (150 mL), respectively in a 500 mL round-bottom flask equipped with a stir bar was mixed with a solution of 2,2'-bipyridine(40 mg, 0.25 mmol, 0.5 equiv.) in $ClCH_2CH_2Cl$ (5 mL). A mixture of Oxone® (3.1 g, 5 mmol, 10 equiv.) and NaOH (290 mg, 5 mmol, 10 equiv.) in water (15 mL) was added periodically over 5 h in equal portions. TLC control (Figure S18). Workup as per general procedure. R_f (silica–CH_2Cl_2/20% hexanes) = 0.48. ^{1}H NMR (400 MHz, $CDCl_3$, δ): 8.82 (d, ^3J = 4.1 Hz, 1H), 8.75–8.81 (m, 3H), 8.68–8.69 (m, 2H). ^{13}C NMR (100 MHz, $CDCl_3$, δ): see ref. [11]. ^{19}F NMR (376 MHz, $CDCl_3$, δ): –137.25 to –137.54 (m, 6F), –138.92 (dd, ^3J = 23.5, ^4J = 7.3 Hz, 2F), –151.83 (dq, ^3J = 39.6, ^4J = 19.9 Hz, 3F), –152.44 (t, ^3J = 20.9 Hz, 1F), –161.55 to –161.76 (m, 6F), -162.29 (td, ^3J = 22.2, ^4J = 7.1 Hz, 2F). UV-vis (CH_2Cl_2) λ_{max} (log ε): 393 (4.52), 417 (5.24), 542 (3.59), 561 (3.87), 606 (4.40) nm. HR-MS (ESI+, 100% CH_3CN, TOF) [9]: *m/z* = 1054.9553 calc'd. for $C_{43}H_7F_{20}N_4O_2Zn$ $[MH]^+$; found 1054.9533.

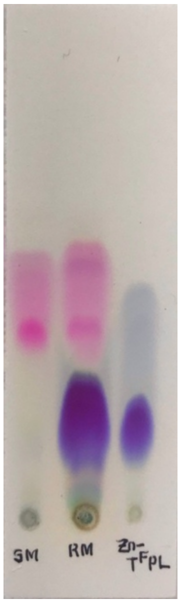

Figure S18. Representative TLC (CH_2Cl_2/25% hexanes) of the reaction mixture (RM) in comparison to the starting materials (SM) and product T^{F}PLZn.

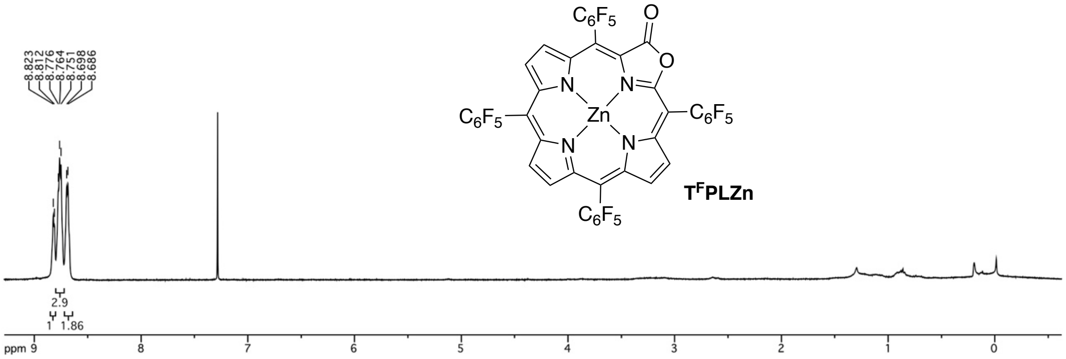

Figure S19. ^{1}H NMR (400 MHz, $CDCl_3$) of T^{F}PLZn.

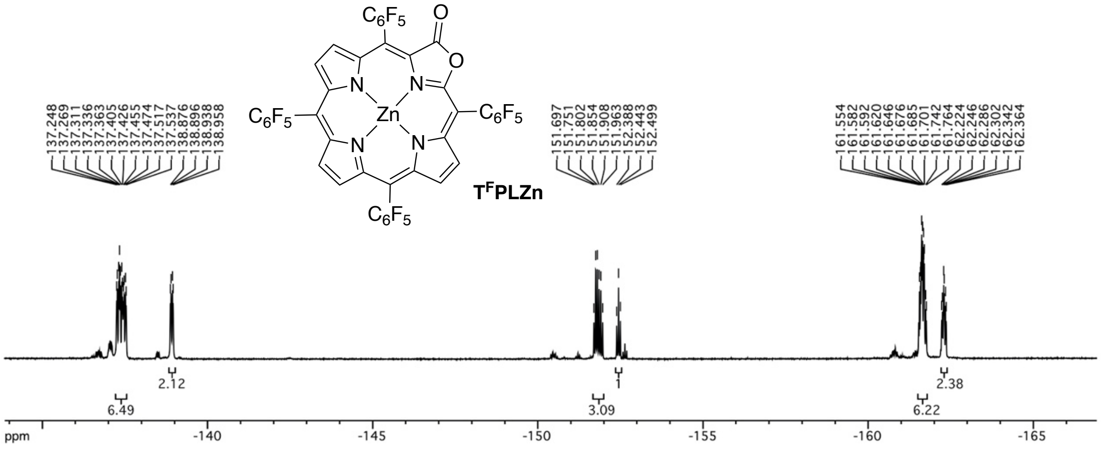

Figure S20. ^{19}F NMR (376 MHz, $CDCl_3$) of T^{F}PLZn.

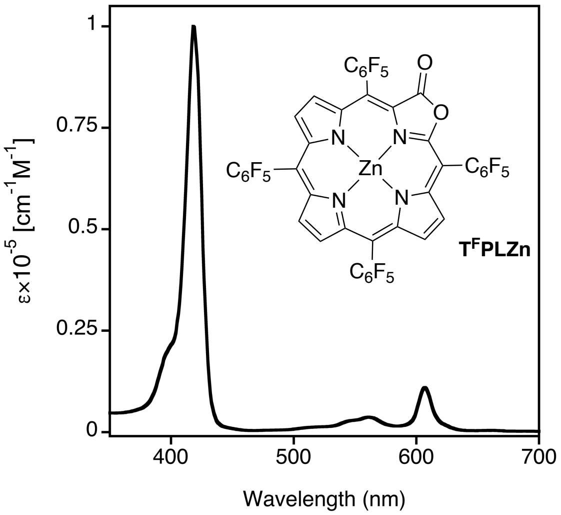

Figure S21. UV-vis spectrum (CH_2Cl_2) of T^{F}PLZn.

Synthesis of [*meso*-Tetrakis(pentafluorophenyl)-2-oxa-3-oxoporphyrinato]platinum(II) (T^FPLPt)

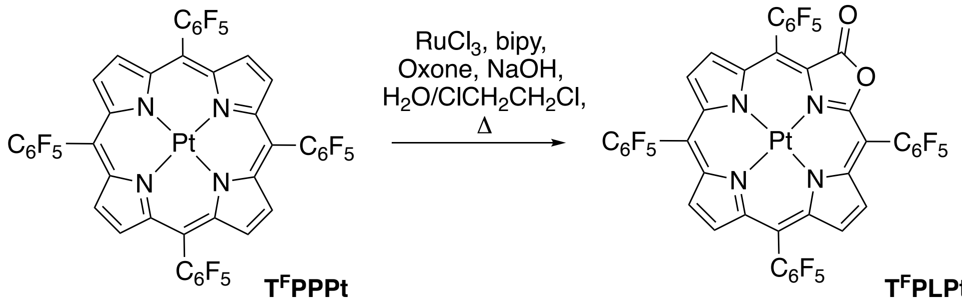

Prepared in 50% yield (191 mg) as a reddish powder according to the general procedure described for T^FPL synthesis using T^FPPPt (100 mg, 0.16 mmol, 1 equiv.) and $RuCl_3$ (16.6 mg, 0.08 mmol, 0.5 equiv.) in $ClCH_2CH_2Cl$ (50 mL) and water (50 mL) in a 250 mL round bottom flask, mixed with a solution of 2,2'-bipyridine (12.5 mg, 0.08 mmol, 0.5 equiv.) in $ClCH_2CH_2Cl$ (1.5 mL). A mixture of Oxone® (243.5 mg, 1.6 mmol, 10 equiv.) and NaOH (64.0 mg, 1.6 mmol, 10 equiv.) in water (5 mL) was added in five portions over 5 h while the solution was refluxed for 8 h. Work up and isolation of product were carried out according to the general procedure. R_f (silica–CH_2Cl_2/20% hexanes) = 0.52. ^1H NMR (400 MHz, $CDCl_3$, δ): 8.68–8.83 (m, 6H) ppm. ^{13}C NMR (100 MHz, $CDCl_3$, D1 = 2.5 s, δ) [11]: 163.2, 148.7, 146.7, 142.7, 141.0, 139.9, 139.3, 138.7, 133.9, 131.6, 130.3, 130.0, 129.9, 129.0, 118.7, 112.0, 108.4, 105.9, 88.0; all *meso*-aryl carbon atoms are broadened, often beyond distinction, because of F-C couplings. ^{19}F NMR (376 MHz, $CDCl_3$, δ): –136.49 to –136.76 (m, 6F), –138.19 (dd, ^3J = 22.8, ^4J = 7.2 Hz, 2F), –150.11 to –150.31 (m, 3F), –150.89 (t, ^3J = 20.9 Hz, 1F), –160.38 to –160.67 (m, 6F), –161.25 (td, ^3J = 21.4, ^4J = 6.3 Hz, 2F). HR-MS (ESI+, 100% CH_3CN, TOF) [9]: *m/z* = 1184.9819 calc'd. for $C_{43}H_6F_{20}N_4O_2Pt$ $[M]^+$; found 1184.9817.

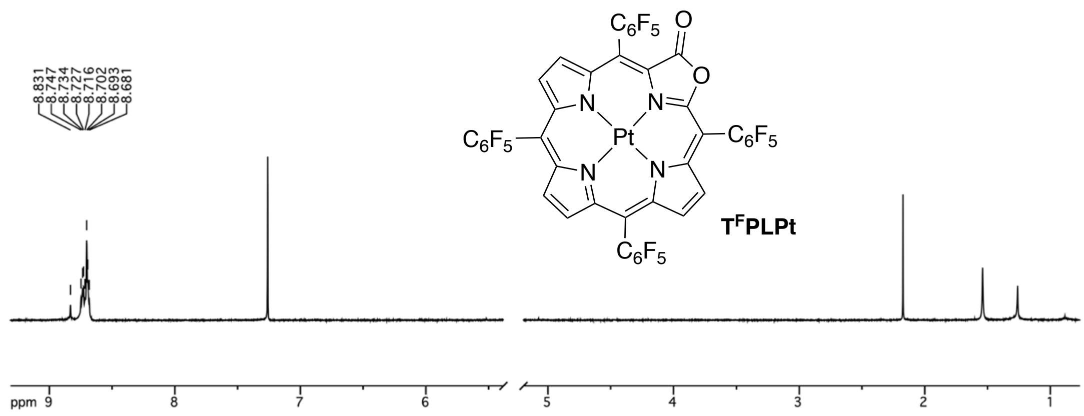

Figure S22. ^{1}H NMR (400 MHz, $CDCl_3$) of T^{F}PLPt.

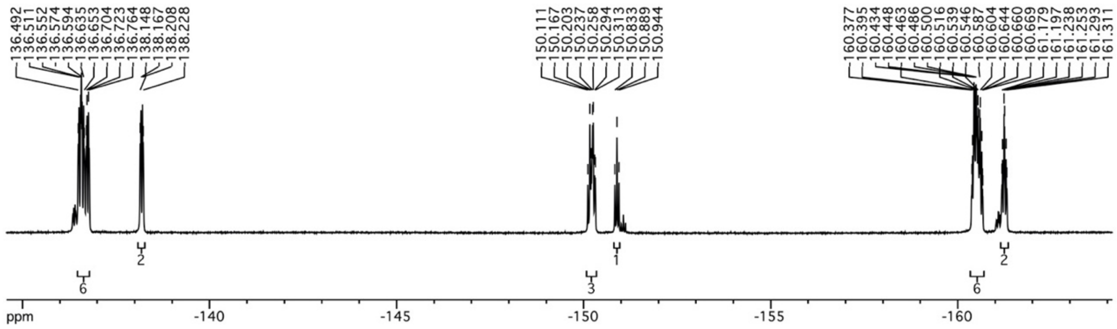

Figure S23. ^{19}F NMR (376 MHz, $CDCl_3$) of T^{F}PLPt.

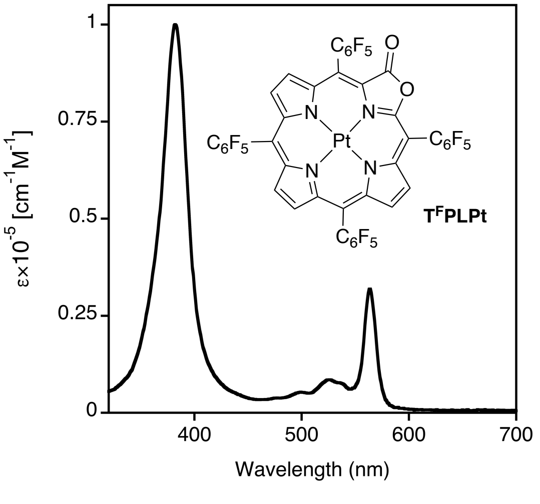

Figure S24. UV-vis spectrum (CH_2Cl_2) of T^{F}PLPt.

ESI References

1. Spellane, P.J.; Gouterman, M.; Antipas, A.; Kim, S.; Liu, Y.C. Electronic Spectra and Four-orbital Energies of Free-base, Zinc, Copper, and Palladium Tetrakis(perfluoro-phenyl)porphyrins. *Inorg. Chem.* **1980**, *19*, 386–391, doi:10.1021/ic50204a021.
2. Available through many standard chemical supply houses but also supply houses specialized on porphyrins, such as Frontier Scientific (https://www.frontiersci.com) or PorphyChem (https://www.porphychem.com).
3. Khalil, G.; Gouterman, M.; Ching, S.; Costin, C.; Coyle, L.; Gouin, S.; Green, E.; Sadilek, M.; Wan, R.; Yearyean, J., et al. Synthesis and Spectroscopic Characterization of Ni, Zn, Pd and Pt Tetra(pentafluorophenyl)porpholactone with Comparison to Mg, Zn, Y, Pd and Pt Metal Complexes of Tetra(pentafluorophenyl)porphine. *J. Porphyrins Phthalocyanines* **2002**, *6*, 135–145, doi:10.1142/S108842460200018X.
4. Adler, A.D.; Longo, F.R.; Finarelli, J.D.; Goldmacher, J.; Assour, J.; Korsakoff, L. A Simplified Synthesis for *meso*-Tetraphenylporphine. *J. Org. Chem.* **1967**, *32*, 476, doi:10.1021/jo01288a053.
5. Furniss, B.S.; Hannaford, A.J; Smith, P.W.G.; Tatchell, A.R. Vogel's Textbook of Practical Organic Chemistry, Longman: Essex, GB, 5th Edition, 1989, p. 549.
6. Brückner, C.; Rettig, S.J.; Dolphin, D. Formation of a *meso*-Tetraphenylsecochlorin and a Homoporphyrin with a Twist. *J. Org. Chem.* **1998**, *63*, 2094–2098, doi:10.1021/jo971156t.
7. Brückner, C.; Ogikubo, J.; McCarthy, J.R.; Akhigbe, J.; Hyland, M.A.; Daddario, P.; Worlinsky, J.L.; Zeller, M.; Engle, J.T.; Ziegler, C.J., et al. *meso*-Arylporpholactones and their Reduction Products. *J. Org. Chem.* **2012**, *77*, 6480–6494, doi:10.1021/jo300963m.
8. Hewage, N.; Zeller, M.; Brückner, C. Oxidations of chromene-annulated chlorins. *Org. Biomol. Chem.* **2017**, *15*, 396–407, doi:10.1039/C6OB02395A.
9. Yu, Y.; Lv, H.; Ke, X.; Yang, B.; Zhang, J.-L. Ruthenium-Catalyzed Oxidation of the Porphyrin ß-ß'-Pyrrolic Ring: A General and Efficient Approach to Porpholactones. *Adv. Synth. Catal.* **2012**, *354*, 3509–3516, doi:10.1002/adsc.201200720.
10. Dean, M.L.; Schmink, J.R.; Leadbeater, N.E.; Brückner, C. Microwave-promoted Insertion of Group 10 Metals into Free Base Porphyrins and Chlorins: Scope and Limitations. *Dalton Trans.* **2008**, 1341–1345, doi:10.1039/b716181f.
11. Khalil, G.E.; Daddario, P.; Lau, K.S.F.; Imtiaz, S.; King, M.; Gouterman, M.; Sidelev, A.; Puran, N.; Ghandehari, M.; Brückner, C. *meso*-Tetraarylporpholactones as High pH Sensors. *Analyst* **2010**, *135*, 2125–2131, doi:10.1039/C0AN00018C.
